# TL-RL-FusionNet: Reinforcement Learning-Guided Residual MLP with Fused CNN Embeddings for Efficient and Adaptive Ransomware Detection

**DOI:** 10.3390/s26154775

**Published:** 2026-07-27

**Authors:** Jannatul Ferdous, Rafiqul Islam, Arash Mahboubi, Md Zahidul Islam

**Affiliations:** 1 School of Computing, Mathematics and Engineering, Charles Sturt University, Wagga Wagga, NSW 2650, Australia; 2 School of Computing, Mathematics and Engineering, Charles Sturt University, Albury, NSW 2640, Australia; 3 School of Professional Studies, University of New South Wales (UNSW), Canberra, ACT 2600, Australia; 4 School of Computing, Mathematics and Engineering, Charles Sturt University, Bathurst, NSW 2795, Australia; 5 AI and Cyber Futures Center, Charles Sturt University, Panorama Avenue, Bathurst, NSW 2795, Australia

**Keywords:** ransomware detection, EfficientNet, InceptionV3, interpretability, MLP, multi-CNN feature fusion, Q-learning, reinforcement learning, sample weighting, transfer learning, cybersecurity, sensor networks

## Abstract

Ransomware detection remains challenging because modern variants exhibit diverse, elusive, and partly benign behaviors and can propagate rapidly across interconnected enterprises and sensor-enabled cyber-physical systems, causing cascading operational failures. These characteristics undermine signature-based and static-detection methods. Although machine learning has improved detection, many approaches still rely on fixed objectives that weight samples uniformly, limiting their adaptation to heterogeneity and overlaps between ransomware and benign activities. To address this challenge, we introduce TL-RL-FusionNet, a reinforcement learning (RL)-guided hybrid framework that combines dual transfer learning (TL) backbones, EfficientNetB0 and InceptionV3, with a lightweight residual multi-Layer perceptron (MLP) classifier. The framework converts sandbox reports into RGB grids, extracts features using frozen CNN backbone networks, and fuses embeddings for classification. Training is guided by a tabular Q-learning sample-weighting agent, formulated as a per-sample bandit over discrete weight actions. To prevent cross-fold information leakage, the Q-table is freshly initialized in each cross-validation fold and updated only using the fold-local training partition, whereas the held-out fold is used for the final evaluation. The framework was evaluated using two datasets. On our dataset, TL-RL-FusionNet achieved the best overall performance on Dataset 1, with 99.20% accuracy, 99.40% recall, and 99.84% AUC. On the public EldeRan benchmark, it achieved 90.36% accuracy using the full dynamic feature space and 92.08% using a Mutual Information-selected compact subset. Paired Wilcoxon tests across five folds were used to assess the RL contribution, while additional grid-order sensitivity analysis showed that the image-based representation remained robust under five random 10 × 10 feature-grid permutations. Interpretability analysis using t-distributed stochastic neighbor embedding (t-SNE) and gradient-weighted class activation mapping feature-grid mapping further showed that the model captured discriminative behavioral patterns. Overall, these results demonstrate that RL-guided sample reweighting improves adaptive ransomware detection while maintaining efficiency and interpretability. The dataset and supporting code are publicly available on GitHub.

## 1. Introduction

Ransomware has emerged as one of the most destructive forms of malware, causing substantial economic losses, disrupting critical infrastructure, and threatening the integrity of data. Unlike traditional malware, ransomware encrypts victims’ data and demands a ransom, often in cryptocurrency, for the decryption key [1]. Modern variants employ polymorphism, obfuscation, and anti-analysis strategies, making their detection using conventional methods increasingly challenging. Additionally, new attack strategies, such as double extortion, in which data are exfiltrated and threatened with public release, have further magnified this threat. The proliferation of Ransomware-as-a-Service (RaaS) [2] has lowered the entry barriers for cybercriminals, and the widespread use of cryptocurrencies facilitates anonymous ransom payments, complicating law enforcement efforts. As attackers continue to innovate, the need for advanced and adaptive ransomware detection mechanisms has become increasingly urgent in recent years.

Ransomware detection typically relies on static and dynamic analyses to identify malicious behavior. Static analysis inspects the code structure without execution, thereby providing a safer and faster detection. However, it faces challenges when dealing with obfuscated or polymorphic ransomware that employs tactics such as packing and encryption. In contrast, dynamic analysis observes the program behavior during execution, enabling the detection of hidden or runtime-triggered actions in the program. However, it is resource-intensive, slow, and susceptible to anti-analysis evasion techniques [1].

Early ransomware detection systems employed traditional machine learning (ML) techniques such as Random Forests (RF), Support Vector Machines (SVM), and Logistic Regression (LR), which learn from features extracted from static or dynamic analyses to classify benign and malicious activities. However, Traditional ML methods require manual feature engineering, making them labor-intensive and prone to bias. These models cannot learn hierarchies from raw data, limiting their adaptability to evolving ransomware [3].

To overcome the reliance on manual features, researchers have turned to deep learning (DL) methods for ransomware detection because of their ability to extract high-level semantic features from raw data and pattern recognition [4,5,6,7]. DL, a subfield of ML, trains computers for automated prediction by employing multilayer artificial neural networks inspired by human brain neurons. These networks consist of interconnected layers that transform input data using nonlinear activation functions (e.g., ReLU, Sigmoid, Tanh), thereby enabling the hierarchical extraction of features from raw input data. The model parameters were optimized through backpropagation, in which gradients were propagated backward to iteratively update the network weights and minimize the errors. By combining nonlinear activation functions with backpropagation-driven optimization, these models achieve superior performance in analyzing high-dimensional data. Convolutional Neural Networks (CNNs) [8,9,10] and Long Short-Term Memory (LSTM) networks [8] have demonstrated potential for enhancing detection accuracy. Additionally, DL techniques have been widely applied in image-based detection pipelines [8,10,11,12]. However, these methods require large, balanced datasets and entail high computational costs, making them less practical for use in real-world applications.

Transfer learning (TL) has emerged as a promising solution to these challenges in recent years. TL leverages knowledge from pretrained CNNs (e.g., ImageNet backbones) to reduce training costs and improve generalization on small datasets. TL is a machine learning technique that allows a model to use previously learned knowledge acquired from one problem domain and apply it to another related domain. By reusing pretrained extractors, TL enhances detection precision, accelerates training, and enables rapid adaptation to new threats. Several studies have employed TL for malware classification, demonstrating strong generalization with limited amounts of data. TL can be applied in two modes: (i) using pretrained models as frozen feature extractors [12,13,14,15] or (ii) full end-to-end fine-tuning CNNs to learn task-specific features [16]. In our previous study [17], we achieved a state-of-the-art accuracy of up to 99.96% through end-to-end fine-tuning of multiple CNN models (ResNet, EfficientNetB0, InceptionV3, Xception, and VGG). Despite these advantages, TL-based ransomware detection still lacks mechanisms for dynamic adaptation to sample weighting and struggles with high resource demands, which are unsuitable for resource-constrained environments.

To address adaptability gaps, researchers have recently explored the use of RL in cybersecurity applications [18,19,20]. RL enables an agent to adjust its actions based on reward feedback, making it suitable for adaptive sample reweighting during the model training. In this study, RL does not explicitly define sample difficulty; instead, it adaptively changes each training sample’s contribution using feedback from fold-local training predictions. This allows the classifier to avoid treating all samples uniformly and adjust its learning focus according to reward-guided weighting. In ransomware detection, such adaptive weighting can help improve learning from behaviorally diverse samples, support more flexible decision boundary formation, and complement CNN-based feature extraction. However, RL alone lacks the representational capacity of deep CNN backbones, motivating the development of a hybrid framework that combines frozen CNN feature fusion with reward-guided sample weighting.

Despite advances in DL and TL for ransomware detection, existing pipelines remain non-adaptive and treat the training samples equally. This leaves models vulnerable to adaptive adversaries such as ransomware, which frequently alter execution strategies to evade detection. In adversarial settings, rare or stealthy behaviors are overshadowed by common patterns [21], reducing the robustness of static or TL-based approaches. RL offers a solution by adaptively adjusting training focus based on feedback, enabling the model to learn from adversary’s evolving strategies [22]. However, prior applications of RL in security have mostly been restricted to static PE features, Android permissions or forensic classification tasks.

To overcome these challenges, we developed TL-RL-FusionNet, a hybrid architecture that integrates TL-based feature fusion with bandit-style RL-guided sample reweighting. In the proposed pipeline, sandbox-generated behavioral JSON reports were converted into structured feature vectors and then transformed into deterministic 10 × 10 RGB feature-grid images. The images were processed using two frozen pretrained CNN backbones, EfficientNetB0 and InceptionV3, for feature extraction. EfficientNetB0 captures fine-grained visual patterns, whereas InceptionV3 captures multi-scale structural patterns. The resulting global-average-pooled embeddings were fused into a single rich representation and classified using the proposed residual MLP and a set of baseline models. Training is guided by a Q-learning agent that adaptively reweights samples within each cross-validation fold independently rather than treating every sample uniformly across the full training run. The proposed RL-guided MLP was benchmarked against six baseline ML/DL classifiers (RF, XGBoost, SVM, LR, ANN, and CNN), each evaluated with and without RL-guided weighting. Two datasets were used for evaluation: Dataset 1, comprising 500 ransomware samples from 25 families and 500 benign applications reflecting recent ransomware and benign execution behaviors; and the public EldeRan benchmark, evaluated using both the full 30,967-feature representation and the Mutual Information-selected top-100 feature subset. The EldeRan evaluation was scoped as an external validation of the proposed architecture rather than a second full baseline comparison. The evaluation also included paired Wilcoxon tests, end-to-end efficiency profiling, grid-order sensitivity analysis, and Grad-CAM mapping back to the behavioral feature grid.

The key contributions of this study are as follows.

- We propose TL-RL-FusionNet, in which training samples are adaptively reweighted by a tabular Q-learning agent acting as a per-sample bandit over five discrete weight actions: {0.25, 0.50, 1.00, 1.25, 1.50}. The Q-table is reset independently every cross-validation fold and updated only from that fold’s own training-partition predictions, so the held-out fold never contributes to reward computation or Q-table updates, preventing cross-fold information leakage while still mitigating overfitting and improving accuracy over static training.
- We pair two frozen pretrained CNN backbones (EfficientNetB0, InceptionV3) with a lightweight residual MLP classifier. Freezing the CNNs avoids backpropagation through millions of parameters and lets embeddings be pre-computed once and reused, while the MLP’s small parameter count keeps training and memory costs low without sacrificing accuracy.
- We benchmark the proposed residual MLP and six baseline classifiers—RF, XGBoost, SVM, LR, ANN, and CNN—under both RL-guided and non-RL configurations, providing a fair assessment of adaptive sample weighting across traditional machine learning, ensemble learning, and deep learning classifiers. We further validate TL-RL-FusionNet on the public EldeRan benchmark using both the full 30,967-feature representation and the Mutual Information-selected top-100 subset, demonstrating its applicability beyond Dataset 1.
- We statistically validate the RL-guided improvement by reporting paired Wilcoxon signed-rank tests on Dataset 1 and EldeRan MI-100 rather than relying on point estimates alone.
- We provide a complete computational efficiency evaluation, including end-to-end runtime and RAM profiling. We also assess interpretability and representation-level robustness through Grad-CAM-to-behavioral-feature mapping and grid-order sensitivity analysis using five randomly permuted 10 × 10 feature-grid arrangements, as reported in Section 4.4.5 and Section 4.4.6.

The remainder of this paper is organized as follows. Section 2 reviews the related work on traditional machine learning (ML), DL, TL, and RL-based ransomware detection. Section 3 details the methodology, including the two datasets, structured-to-image conversion, dual-CNN feature fusion, RL-guided sample reweighting, and evaluated classifiers. Section 4 presents the experimental results, covering the cross-validation performance with and without RL, computational efficiency, interpretability analysis, external validation on the EldeRan benchmark, and a comparison with existing methods. Section 5 discusses the findings and their limitations. Finally, Section 6 concludes the paper and provides directions for future work.

## 2. Related Works

This section reviews prior research in three areas relevant to the proposed framework: (1) traditional machine learning and deep learning for ransomware detection, (2) transfer learning and multi-CNN feature fusion, and (3) reinforcement learning for adaptive detection. Table 1 provides a comparative synthesis of representative studies covering feature representations, learning architectures, datasets, performance metrics, strengths, and limitations.

### 2.1. Traditional Machine Learning and Deep Learning for Ransomware Detection

The increasing complexity and evasion capability of modern ransomware have driven a shift from signature-based detection to data-driven approaches based on static features, dynamic behavioral traces, and visual feature representations. Static-feature-based studies typically analyze executable structures such as PE headers. Manavi and Hamzeh proposed an LSTM model using PE header byte representations, achieving 93.25% accuracy on 1000 ransomware and 1000 benign samples [23]. They later converted PE headers into zigzag grayscale images and trained a CNN model, achieving an accuracy of 93.33% [24]. However, this method does not explicitly address the generalization of unseen ransomware families. Similarly, Moreira et al. [10] transformed PE headers into color images and classified them using Xception CNN, achieving 98.20% accuracy; however, static representations may be bypassed by packing, obfuscation, and polymorphic ransomware. They emphasized the importance of execution features for understanding advanced ransomware attacks.

Dynamic analysis methods address this limitation by considering the runtime behavior. Sgandurra et al. [3] proposed EldeRan, a Cuckoo-based framework using Mutual Information and regularized Logistic Regression (RLR), achieving 0.995 AUC and 96.3% detection on 582 ransomware and 942 benign samples. However, its Windows XP/Cuckoo telemetry and conventional ML design may limit its applicability to modern adaptive ransomware attacks. Qin et al. [25] used an improved TextCNN on API-call sequences, achieving 95.9% accuracy. Similarly, XRan [26] leveraged CNNs on dynamic features (API calls, DLLs, mutexes) integrated with XAI methods such as LIME and SHAP, achieving a true positive rate of 99.4% at the cost of high computational overhead from dynamic feature extraction and explanation generation. SwiftR [27] applies hierarchical neural networks (HNN) across multiple platforms and demonstrates robust cross-platform detection performance.

Collectively, these studies demonstrate the effectiveness of ML and DL in ransomware detection; however, limitations remain in terms of computational efficiency, dataset balance, interpretability, and adaptability to evolving ransomware behaviors.

### 2.2. Transfer Learning and Multi-CNN Feature Fusion

TL is effective in contexts where domain-specific training data are scarce. Pretrained CNNs, such as VGG16, ResNet50, EfficientNet, and InceptionV3, originally trained on large-scale image datasets (e.g., ImageNet), can be adapted for malware image classification using techniques such as fine-tuning and feature extraction.

For example, Almomani et al. [28] introduced E2E-RDS, a vision-based ransomware detection system that transforms binaries into two-dimensional images, and evaluated 19 CNN models. The fine-tuned ResNet50 achieved 99.5% accuracy, outperforming the static ML baselines. Rezende et al. [29] used TL with ResNet-50 on grayscale byte plots, reporting 98.62% accuracy across 9339 samples from 25 families without addressing imbalanced datasets or new malware variants. Huang et al. [30] developed a hybrid model combining static and dynamic image representations, with VGG16 achieving 94.7% on hybrid data. However, this study did not evaluate the scalability or resource efficiency of these models. In our previous work [17], we developed a ransomware detection framework that converts sandbox features into grayscale and color images and applied fine-tuned CNNs, including ResNet50, EfficientNetB0, InceptionV3, Xception, VGG16, and VGG19 models. ResNet50 achieved 99.96% accuracy for 500 ransomware and 500 benign samples. However, fine-tuning requires a longer training time and memory, which may limit its practicality in resource-constrained environments.

Subsequent studies have demonstrated the benefits of multi-backbone fusions. Shah et al. [13] introduced a dual-stage DWT with a modified DenseNet-121 for feature extraction and machine learning classifiers, with XGBoost achieving 98.58% accuracy. The method optimized TL for grayscale inputs but lacked evaluation on unseen samples. Rustam et al. [14] proposed a bimodal framework using VGG16 and ResNet50 as feature extractors, with traditional classifiers applied to fused features; their Bi-SVC achieved 100% accuracy on the Malimg dataset, but the work was limited to a single benchmark dataset, raising concerns of overfitting. Rezende et al. [15] used VGG16 and SVM to classify malware families with 92.97% accuracy but underperformed newer ensembles. Kumar and Panda [12] proposed an SDIF-CNN using VGG16, VGG19, ResNet50, and InceptionV3 as feature extractors, with an MLP classifier achieving 98.55% accuracy on Malimg and strong generalization on packed malware. Vasan et al. [11] developed IMCEC, an ensemble of pretrained CNNs with SVM and Softmax classifiers, achieving 99.5% accuracy on Malimg and showing resilience to obfuscation.

Collectively, these studies underscore TL’s ability of TL to deliver high accuracy and reduce manual feature engineering. However, most existing methods focus on single datasets and static visualizations without adaptive-sample prioritization. Moreover, while accuracy is emphasized, efficiency (time, RAM), and model interpretability have been less explored.

### 2.3. Reinforcement Learning for Adaptive Ransomware Detection

RL has recently been explored for ransomware detection because of its adaptability to evolving threats. Unlike conventional ML, RL agents optimize policies via reward-driven interactions, making them suitable for dynamic threat environments.

Deng et al. [18] proposed an early ransomware detection framework that applied Double Deep Q-Learning (DDQN) to portable executable (PE) header features. This method achieved fast classification but was inherently limited by its reliance on static PE headers, which are susceptible to packing and obfuscation. Kumar et al. [19] integrated RL with Bayesian networks and dynamic embeddings to strengthen resilience modelling across system configurations and vulnerabilities. Although effective for adaptive defense, this approach focuses on resilience prediction rather than direct classification of ransomware. Jeremiah et al. [20] used Deep Reinforcement Learning (A2C, DQN, DDQN, PPO) on Android permissions and network traffic. Their A2C and DDQN models surpassed traditional ML baselines in terms of accuracy and speed, although they only considered static features rather than behavioral representation. CyberForce uses federated reinforcement learning (FRL) for IoT malware mitigation, achieving 98% accuracy and reducing the training time by 67% compared with centralized methods [31]. While RL shows promise in adaptability and performance, studies are limited to static PE features and Android contexts, with minimal focus on image-based ransomware or accuracy-efficiency optimization.

Overall, prior studies have demonstrated strong progress in ransomware detection, particularly through dynamic behavioral features, image-based representations, and pretrained CNNs. However, existing studies have several limitations. Traditional ML methods depend heavily on handcrafted features, DL models often require large datasets and substantial computation, and TL-based pipelines usually treat all training samples uniformly. Existing RL-based security studies provide adaptive learning mechanisms but are mostly limited to static PE features, Android attributes or resilience modelling. These gaps motivated the development of TL-RL-FusionNet, which combines sandbox-derived behavioral feature-grid images, frozen dual-CNN feature fusion, and fold-local RL-guided adaptive sample weighting.

## 3. Methodology

**Figure 1:**
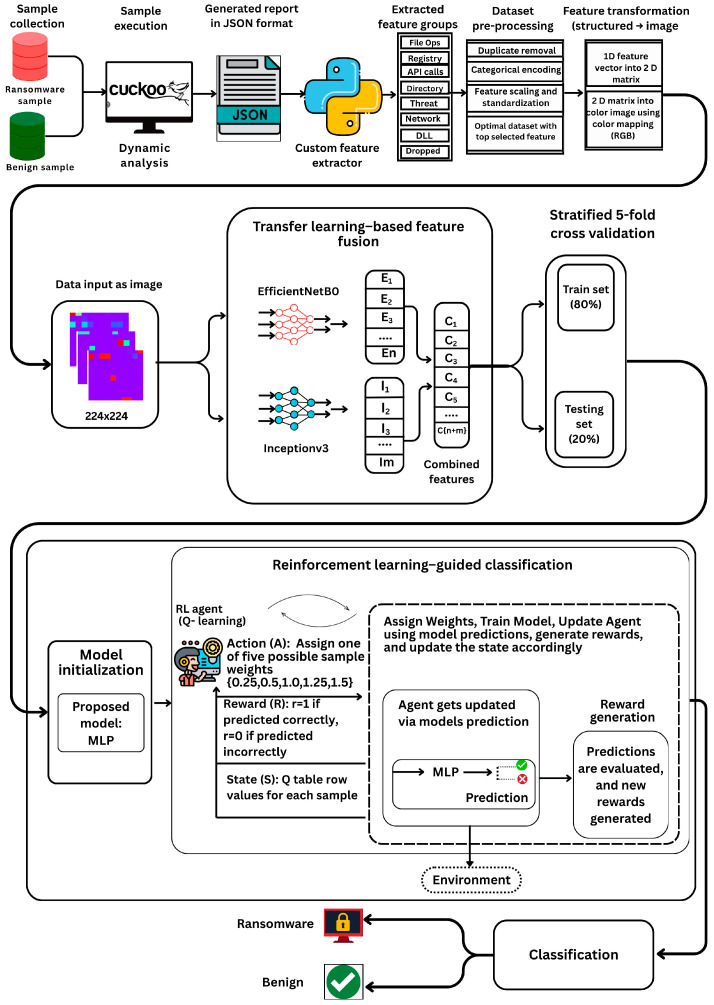
End-to-end TL-RL-FusionNet workflow for ransomware detection.

This section presents the methodology used to develop and evaluate TL-RL-FusionNet. The framework comprises five main stages: (i) dataset description and preprocessing, (ii) structured-to-image feature conversion, (iii) dual-CNN feature extraction and fusion, (iv) reinforcement learning-guided sample weighting, and (v) final classification. Figure 1 illustrates the overall architecture and operational workflow of our proposed framework.

### 3.1. Dataset Description

#### 3.1.1. Dataset Overview

**Dataset 1:** This study was primarily evaluated using Dataset 1, a custom behavioral ransomware dataset created in our previous study [17]. Dataset 1 contains 1000 Windows executable samples, comprising 500 ransomware and 500 benign application samples. The ransomware subset covered 25 active ransomware families, whereas the benign subset contained legitimate Windows applications collected from trusted software repositories. Each sample was executed in a controlled sandbox environment, and the generated behavioral reports were converted into fixed-length feature vectors for machine learning-based ransomware detection.

**Dataset 2: EldeRan dataset.** In addition to Dataset 1, the public EldeRan [3] benchmark was used as an external validation dataset for the proposed model. EldeRan is a Cuckoo-based dynamic analysis dataset generated in a Windows XP sandbox from ransomware and goodware executions. The original dataset contained 582 ransomware samples from 11 families and 942 benign applications, with host-based dynamic features extracted from API calls, registry operations, file system activity, directory operations, dropped files, file extensions, and embedded strings. In our preprocessing, the dataset was balanced to 581 ransomware and 581 benign samples, producing 1162 total instances. Two configurations were evaluated: the complete 30,967-feature representation and a compact 100-feature subset selected using Mutual Information. For compatibility with TL-RL-FusionNet, the full feature vector was reshaped into a near-square grid and resized to (224 × 224) RGB images, while the top-100 features were arranged into a (10 × 10) grid and resized similarly. Both configurations were evaluated independently using the unchanged TL-RL-FusionNet architecture under stratified five-fold cross-validation.

#### 3.1.2. Sample Collection and Selection Criteria

The ransomware samples for Dataset 1 were collected from MalwareBazaar [32] and VirusShare [33] from 25 active ransomware families. These repositories were selected because they provide access to real-world malware binaries and metadata that are useful for family verification. Benign samples were obtained from trusted software sources, including SnapFiles [34], PortableApps.com [35], and GitHub-hosted Windows-executable repositories [36]. These benign sources were selected to include diverse legitimate software behaviors, such as file access, installation activity, process creation, registry interaction, and network communication.

Ransomware families were selected based on the following criteria:

1. Documented prevalence and organizational impact in threat intelligence reports from 2019 [37], 2020 [38], 2021 [39], 2022 [40], 2023 [41], and 2024 [42].
2. Verification across at least two independent reports from reputable cybersecurity vendors.

This selection method aimed to identify prominent ransomware families, such as LockBit, MedusaLocker, BlackCat, Phobos, and Conti, to ensure that the dataset included a representative coverage of advanced and adaptive threats. Prioritizing these high-impact variants enhances the understanding of critical ransomware behaviors and increases the relevance of the dataset for the detection models.

Individual ransomware samples within each family were selected following the procedure described in [10], with three criteria applied based on VirusTotal vendor engine detection. A ransomware sample was included if it met all the following criteria.

1. At least 45 antivirus engines on VirusTotal classified these files as malicious.
2. At least 15 antivirus engines specifically labelled the files as ransomware.
3. The majority of engines, or at least ten, identified the file as belonging to the same ransomware family.

Samples that did not satisfy these criteria were excluded from the curated dataset rather than being relabeled or manually forced into a family. This filtering reduced the risk of label noise, ambiguous family assignment, and accidental inclusion of non-ransomware malwares. It is important to note that VirusTotal was used solely for sample curation and family label verification. Its labels, detection counts, confidence indicators, reputation scores, and antivirus outputs were excluded from the feature space and not used for training or inference. Consequently, the model performance reflected behavioral discriminability rather than VirusTotal-derived label proxies. Table 2 reports the final distribution of the 25 ransomware families included in Dataset 1.

#### 3.1.3. Sample Execution and Feature Extraction

Each sample was executed in an isolated Cuckoo Sandbox environment [43], and reports were generated in JSON format, which documented both the ransomware attack lifecycle and the normal footprint of benign software, forming the basis for feature extraction. The purpose of sandbox execution was to observe the runtime behavior rather than relying on static executable properties alone. This is important because modern ransomware often uses packing, obfuscation, staged execution, privilege escalation, anti-analysis behavior, shadow copy deletion, and delayed payload activation. Therefore, behavioral execution traces provide richer evidence for distinguishing ransomware from benign software than static metadata alone.

A custom Python-based pipeline was used to parse these reports into structured fixed-length feature vectors. From each sample, 100 highly discriminative behavioral indicators were selected, spanning file system changes, registry modifications, network activity, process and memory operations, API call patterns, and anti-analysis artifacts. The selected features were cross-referenced with MITRE ATT&CK tactics and validated against threat reports from Sophos, CrowdStrike, and Trend Micro (2019–2024). This curated set ensures technical depth, practical relevance, and improved model detection performance across various ransomware families.

#### 3.1.4. Data Preprocessing

Data preprocessing was applied to ensure data quality, numerical consistency, leakage control, and compatibility with the TL-RL-FusionNet. Duplicate records from repeated executions were removed, and non-numeric fields were converted into numerical form. Boolean indicators were encoded as 1/0, string attributes were mapped using length- or frequency-based values, and list-based attributes such as dropped files, DNS requests, contacted hosts, and command-line entries were represented by element counts.

Feature scaling was applied to improve the classifier’s stability. To avoid preprocessing leakage, feature normalization was performed for each cross-validation fold. The scaler was fitted only on the training partition of the current fold and then applied to the corresponding held-out fold. This procedure was applied identically for Dataset 1 and both EldeRan configurations, mirroring the fold-local isolation already enforced for the Q-table described in Section 3.4.1. The final Dataset 1 representation retained 100 discriminative behavioral features selected through feature selection and validated using the MITRE ATT&CK mapping and threat intelligence reports. These features describe observable runtime behaviors across the filesystem, registry, process, network, API/DLL, and anti-analysis domains.

The 100-feature representation used in this study was revised from the schema originally reported in a previous study [17]. To reduce leakage and detection-rule bias, all VirusTotal-derived attributes, including detection counts, antivirus outputs, reputation scores, and vendor labels, were excluded from the feature space. Aggregate detection-count features such as yara_markcount and total_ttp_count were also removed because they may encode detection-rule knowledge rather than raw behavior. The final feature names describe observable runtime actions, ensuring that the TL-RL-FusionNet learns behavioral evidence rather than label-derived shortcuts.

### 3.2. Conversion of Structured Features into Image

To enable CNN-based transfer learning, structured one-dimensional feature vectors from both datasets were transformed into image representations using a single, unified conversion procedure (generate_images.py, released alongside this study on GitHub), applied consistently across Dataset 1 and both reported EldeRan configurations.

For Dataset 1, the selected 100 behavioral features were arranged into a (10 × 10) grid and resized to (224 × 224) RGB images. For Dataset 2, two EldeRan configurations were prepared: the complete 30,967-feature vector was reshaped into a near-square grid and resized to (224 × 224) RGB images, while the MI-selected top-100 features were arranged into a (10 × 10) grid and resized similarly.

Because the same conversion procedure, color encoding, and resize method were applied identically across all three configurations, differences between them arose from the grid resolution and feature dimensionality rather than from any change in the underlying structured-to-image transformation itself. This unified procedure allows EfficientNetB0 and InceptionV3 to extract transferable deep representations from both datasets without modifying the TL-RL-FusionNet architecture.

### 3.3. Transfer Learning for Feature Fusion from Image Data

Two pretrained CNNs, EfficientNetB0 and InceptionV3, were used as the frozen feature extractors. Both models were pretrained on ImageNet, and their convolutional layers were frozen to preserve the generalized image representation capabilities while reducing the training cost. The same frozen dual-backbone configuration was applied to Datasets 1 and 2 to ensure that the proposed architecture remained unchanged across the primary and external benchmark evaluations. Their selection was driven by two factors.

1. Complementary strengths: EfficientNetB0 applies compound scaling to balance the depth, width, and input resolution efficiently, making it suitable for extracting fine-grained behavioral patterns at a relatively low computational expense. InceptionV3 uses inception modules to capture multi-scale spatial patterns, complementing the local-detail representation of EfficientNetB0. For each input image, both CNNs generated global average-pooled embeddings that were concatenated into a single fused feature vector. This fusion combines complementary feature perspectives and provides a richer representation for downstream classification.

2. Proven success in ransomware detection: Both models provide a favorable accuracy-efficiency trade-off for ransomware detection [11,12]. In our previous study [17], ResNet50 achieved the highest accuracy (99.96%), followed by EfficientNetB0 (99.78%) and InceptionV3 (97.84%). However, ResNet50 required a higher training cost, whereas EfficientNetB0 and InceptionV3 offered strong performances with lower computational demands. Therefore, these two backbones were selected as practical frozen feature extractors for TL-RL-FusionNet, allowing transferable embeddings to be reused across datasets while avoiding full CNN fine-tuning.

### 3.4. Reinforcement Learning-Based Sample Weighting

RL is a machine learning paradigm in which an agent learns to make sequential decisions through trial-and-error interactions with an environment to maximize the cumulative reward. Unlike supervised learning, RL does not rely solely on labelled datasets; instead, it learns optimal strategies through feedback from the environment [18].

In ransomware detection, RL can guide a model to emphasize the most informative samples during training phase. Instead of treating all data points equally, the RL agent assigns varying weights to the training samples, enabling the classifier to focus on examples that are more influential in improving the decision boundaries. This targeted weighting helps the model learn patterns that are more representative of ransomware and benign behaviors, even when these patterns are subtle or imbalanced in the dataset.

**Figure 2:**
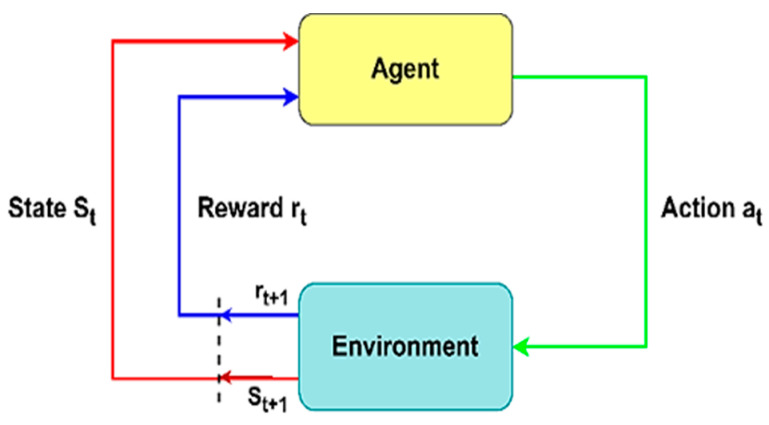
Basic Reinforcement Learning components and information flow between the Agent and Environment, showing the sequence of state observation, action selection, reward feedback, and state transition.

The general interaction loop between the RL components is illustrated in Figure 2, which highlights the flow of states, actions and rewards [44]. At each time step t, the agent observes the current state St, selects an action At, receives a reward Rt from the environment based on the classification performance, and transitions to the next state, St + 1. Over time, the agent optimizes its decision policy by balancing the exploration of new weighting strategies with the exploitation of those that have high-reward actions.

#### 3.4.1. Fold-Local Bandit-Style Sample Weighting Formulation

In this study, the adaptive weighting module was formulated as a tabular bandit-style sample-weighting mechanism rather than a full Markov decision process. Each training sample is represented by an independent row in the Q-table, and the agent selects one of five discrete sample-weight actions, A = {0.25,0.50,1.00,1.25,1.50}. The selected action determines the contribution of the sample to the weighted cross-entropy loss used to train the residual MLP. Because sample states are fixed and no transition dynamics between states are modelled, the method is best interpreted as reward-guided adaptive sample weighting rather than as a generalizing RL policy over unseen states. The components of the proposed adaptive weighting mechanism are defined as follows.

Agent: A fold-local Q-table-based adaptive-weighting module.

- Environment: The residual MLP training process within the current cross-validation fold.
- State: A training sample index represented by a row in the Q-table.
- Action: A discrete weight multiplier selected from {0.25, 0.50, 1.00, 1.25, 1.50}.
- Reward: $r_{i}=1$ if the sample is correctly classified by the fold-local probe model and $r_{i}=0$ otherwise.
- Policy update: The Q-values are updated using only the training samples from the current fold. The held-out test fold is excluded from all reward computations and Q-table updates.

#### 3.4.2. Integration into the Model Training Pipeline

The adaptive weighting mechanism was applied within the stratified cross-validation framework described in Section 4.3. In each fold, a new Q-table was initialized, preliminary weights were assigned to the training samples, and a probe residual MLP was trained on the weighted training partition. Predictions from this probe model on the same training partition were then used to update the fold-local Q-table.

The updated Q-table was used to assign the final sample weights, and a fresh residual MLP was trained using these weights. The held-out fold was used only after the final training for performance evaluation and was never used for reward computation, Q-table updates, sample-weight assignment, or model optimization. The Q-table was discarded after each fold.

This fold-local design prevents information transfer across folds and ensures that the RL-guided and non-RL configurations are compared using identical training/test splits under strict separation.

The complete fold-level training procedure is summarized in Algorithm 1.
**Algorithm 1.** Corrected Fold-Local Bandit-Style Sample Weighting**Input:**Training partition $T_{f}$ for fold f; held-out partition $V_{f}$; labels y; action setA = {0.25, 0.50, 1.00, 1.25, 1.50}.learning rate α, discount factor γ, and exploration rate ε.**Output:**Fold-local sample weights $w_{f}$, trained residual MLP for fold f.1. Initialize a new fold-local Q-table $Q_{f}\in R^{N\times |A|}$ with all values set to zero.2. For each training sample ${i\in T}_{f}$, select an action index $a_{i}$ using an ε-greedy policy.3. Assign the preliminary sample weight $w_{i}$ = A[$a_{i}$].4. Train a probe residual MLP on $T_{f}$ using the preliminary weights.5. Predict the labels of the same fold-local training samples $T_{f}$ using the probe model.6. Compute the reward:$r_{i}=\left\{\begin{array}{c} 1\;if\;{\hat{y}}_{i}=y_{i},\\ 0\;\mathrm{otherwise}. \end{array}\right.$.7. Update $Q_{f}(i,a_{i})$ using the observed reward.8. Reassign final fold-local training weights using the updated $Q_{f}$.9. Train a fresh residual MLP on $T_{f}$ using the final weights.10. Evaluate the final model once on the held-out fold $V_{f}$.11. Discard $Q_{f}$ before proceeding to the next fold.**Note:** The held-out fold $V_{f}$ is not used in Steps 1–9. It is used only in Step 10 for the final performance estimation. No Q-values, rewards, selected actions, sample weights or prediction outcomes were transferred across the folds.

### 3.5. Classifiers Used in This Study

Although the residual MLP is the core classifier of TL-RL-FusionNet, its performance was benchmarked against six additional classifiers: ANN, CNN, LR, RF, XGBoost, and support vector machine (SVM). These models were selected to represent different learning paradigms, including linear learning, kernel-based learning, ensemble learning, gradient boosting, and lightweight deep learning. All classifiers were trained and evaluated using the same fused EfficientNetB0–InceptionV3 embedding representations. To assess the contribution of adaptive sample weighting, each classifier was evaluated under both non-RL and fold-local RL-guided configurations using identical stratified cross-validation.

#### 3.5.1. Residual MLP Model Development (Proposed)

**Figure 3:**
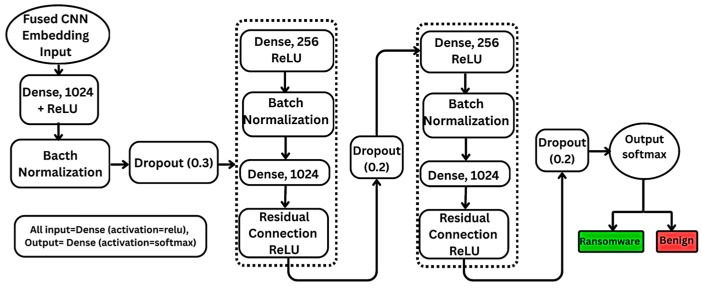
Architecture of the residual MLP classifier used in TL-RL-FusionNet. The classifier receives the fused 3328-dimensional EfficientNetB0–InceptionV3 embedding and applies an initial dense layer followed by two residual blocks and a final softmax layer for ransomware–benign classification.

The proposed residual MLP is a feedforward classifier designed to operate on fixed-length fused embeddings extracted from the frozen EfficientNetB0 and InceptionV3 backbones. EfficientNetB0 generates a 1280-dimensional global average pooled embedding, whereas InceptionV3 generates a 2048-dimensional global average pooled embedding. These two vectors were concatenated to form a 3328-dimensional input representation for the residual MLP. The network began with a dense layer containing 1024 hidden units, followed by batch normalization and dropout at 0.3 dropout rate. The core architecture contains two residual blocks. Each residual block consisted of a 256-unit dense layer with ReLU activation, batch normalization, a 1024-unit dense layer, residual addition, ReLU activation, and a dropout of 0.2. The final layer uses a two-unit softmax classifier for binary ransomware and benign classification. The residual MLP architecture is shown in Figure 3.

The residual design improves the gradient propagation by allowing each block to learn a residual transformation instead of a complete mapping. This supports stable training on compact CNN-derived behavioral embeddings while avoiding the additional computational cost of fine-tuning CNN backbones.

The complete architectural values are summarized in Table 3, and the training configuration and hyperparameters are listed in Table 4.

**Residual MLP Forward Computation and Training Objective**

The classification process of the proposed lightweight MLP is detailed in the following section, including its linear transformations, nonlinear activations, residual connections, normalization, and regularization strategies.

Let $x\in R^{3328}$ denote the fused CNN embedding for a sample. The initial dense transformation is defined as

(1) z=Wx+b

where *W* and *b* are the trainable weight matrix and bias vectors, respectively. The ReLU activation is computed as follows:

(2) $$ReLU\left(z\right)=max(0,\;z)$$

Therefore, the first hidden representation is

(3) $$h_{0}={Dropout}_{0.3}(BatchNorm(ReLU(W_{0}x+b_{0})))$$

Each residual block refines the hidden representation through a Dense–BatchNorm–Dense transformation with identity addition:

(4) $$h_{k+1}={Dropout}_{0.2}\;(ReLU(F\;(h_{k},\;W_{k})+\;h_{k}))$$

where $F\;(h_{k},\;W_{k})$ denotes the transformation inside the residual block. The final output layer produces class probabilities using softmax as follows:

(5) $${\hat{y}}_{c}=\frac{exp(z_{c})}{\sum_{j=1}^{C}exp(z_{j})},\;C=2$$

For the non-RL configuration, the model was trained using categorical cross-entropy with label smoothing and uniform sample contribution. For the RL-guided configuration, the fold-local weighted loss is

(6) $$L_{RL}^{\left(f\right)}=\frac{\sum_{i\epsilon T_{f}}w_{i}l(y_{i\;},\;{\hat{y}}_{i})}{\sum_{i\epsilon T_{f}}w_{i}}$$

where $T_{f}$ is the training partition of fold *f,* $w_{i}$ is the fold-local RL-assigned sample weight, and $l(y_{i\;},\;{\hat{y}}_{i})$ is the label-smoothed categorical cross-entropy loss. The optimized parameters were obtained as follows:

(7) $${\theta \;*\;}_{f}={argmin}_{\theta }L_{RL}^{\left(f\right)}$$

Algorithm 2 summarizes the forward classification pipeline of the residual MLP from the feature vector input to the probability estimation.
**Algorithm 2.** Residual MLP Classifier**Input:** Fused CNN embedding $x\in R^{3328}$
**Output:** Softmax probability vector $\hat{y}\;\in \;R^{2}$
**Input transformation**$h_{0}\leftarrow {Dropout}_{0.3}[BatchNorm(ReLU({Dense}_{1024}(x)+b_{0})))]$2.**Residual blocks**For each residual block k = 1,2:$res\leftarrow h_{k-1}$$h\leftarrow {Dense}_{256}(h_{k-1})\to ReLU\to BatchNorm\to {Dense}_{1024}$$h_{k}\leftarrow {Dropout}_{0.2}[ReLU(h+res)]$3.**Output layer**$\hat{y}\leftarrow Softmax({Dense}_{2}(h_{2}))$4.**Training configuration**The residual MLP was trained using Adam with cosine annealing, categorical cross-entropy with label smoothing of 0.1, a batch size of 32, and 25 epochs under stratified five-fold cross-validation.

#### 3.5.2. Baseline Models

To provide a comprehensive comparison, TL-RL-FusionNet was benchmarked against six baseline classifiers (RF, XGBoost, SVM, LR, ANN, and CNN) trained on the same fused EfficientNetB0–InceptionV3 embeddings. Each model in the benchmark set was carefully selected based on its relevance in the ransomware classification literature, compatibility with the transformed feature space (CNN-extracted high-level embeddings), and ability to provide complementary perspectives on the learning behavior under RL-based sample weighting.

Tree-based ensembles (RF, XGBoost) are well-suited because they handle high-dimensional, structured embeddings efficiently and provide robustness against feature noise, enabling the capture of nonlinear interactions among the CNN-extracted features. Margin-based and linear learners (SVM, LR) offer complementary perspectives by leveraging the inherent separability of CNN embeddings. While SVM exploits high-dimensional margins with kernel functions, LR provides a linear baseline to test whether the features are linearly separable. Neural networks (ANN and CNN) extend the evaluation to deep learning paradigms. ANN leverage dense layers to capture higher-order dependencies within concatenated embeddings, whereas CNN assess whether spatial locality from image representations is preserved and beneficial after feature fusion.

These baselines were selected to assess whether the proposed residual MLP and fold-local adaptive weighting provided benefits beyond those of conventional classifiers.

For a fair comparison, all classifiers used the same stratified five-fold cross-validation splits. In the non-RL setting, the models were trained with uniform sample contributions. In the RL-guided setting, the same corrected fold-local sample-weighting mechanism was applied, where supported. For the deep learning models, the weights were applied through weighted loss during model fitting. For classical machine learning models, the generated weights were passed through the sample_weight interface of the estimator, where supported.

The working processes of these classifiers, including training and prediction, are described in the following sections.

Random Forest (RF): Random Forest is an ensemble learning method that constructs multiple decision trees using bootstrapped subsets of the training data and random subsets of features at each split. The final prediction is obtained by aggregating the outputs of all trees through majority voting, thereby reducing the variance and enhancing the robustness against overfitting [45].

The prediction of a Random Forest with M trees can be expressed as

(8) $$\hat{y}=mode\left\{h_{m}\left(x\right)\;\right|m=1,\;2,\ldots ,M\}$$

where *h*_*m*_(*x*) is the prediction of the *mth* decision tree, and

By aggregating multiple weak learners, Random Forest achieves stable performance on high-dimensional CNN-derived features, making it effective for classifying ransomware, as it leverages the robustness of ensemble methods to improve detection accuracy.

Support Vector Machine (SVM): The SVM classifier is a supervised ML model that divides an n-dimensional space-based representation of the data into two classes using a selected boundary, known as a hyperplane. This hyperplane maximizes the margin between the two regions or classes (in our experiment, ransomware or benign software). The maximal margin is defined as the largest distance between examples of both classes, computed from the distance between their closest instances (called supporting vectors) [4]. It is designed to maximize class separability while minimizing the classification error by using slack variables.

Formally, the optimal hyperplane is represented by a vector *w* and a scalar *m* such that the inner products of *w* with vectors *ϕ*(*x*) from the two classes are divided by an interval between −1 and +1, subject to b. Prediction is given by:

(9) $$\hat{y}=sign(w^{T}\emptyset \left(x\right)+b)$$

where $w^{T}$ denotes the transpose of weight vector *w.*

The ability of SVM to handle high-dimensional fused features makes it a strong baseline for distinguishing ransomware from benign classes, particularly when dual-CNN embedding increases feature separability.

Extreme Gradient Boosting (XGBoost): XGBoost is an advanced ensemble learning technique that enhances the traditional gradient boosting algorithm by integrating strong regularization terms to prevent overfitting. Its scalability and efficiency make it particularly effective for high-dimensional feature extraction. XGBoost builds models sequentially, where each new learner attempts to reduce the residual errors of the previous ensemble [46].

The learning objective combines data fitting loss with a regularization penalty:

(10) $$L\left(\theta \right)=\sum_{i=1}^{n}l\left(y_{i},\;{\hat{y}}_{i}\right)+\sum_{k=1}^{K}\Omega (f_{k})$$

Here, *L*(*θ*) represents the overall regularized objective, $l\left(y_{i},\;{\hat{y}}_{i}\right)$ is the loss between the true and predicted labels, *K* is the total number of boosting trees, and $\Omega (f_{k})$ is the regularization component that penalizes model complexity. The regularization function is defined as

(11) $$\Omega \left(f_{k}\right)=\gamma^{T}+\frac{1}{2}\lambda {\left|\left|w\right|\right|}^{2}$$

where *T* is the number of leaves in the decision tree, *γ* is the regularization coefficient that penalizes the number of leaves, and λ is the regularization parameter applied to the leaf weights *w*.

The final prediction was obtained by aggregating the outputs of all the boosted trees:

(12) $$\hat{y}=\;\sum_{m=0}^{M}\eta h_{m}\left(x\right)$$

where *M* denotes the total number of trees, $h_{m}\left(x\right)$ is the prediction from the *mth* tree, and *η* is the learning rate that controls the contribution of each tree.

XGBoost constructs a robust model that effectively minimizes the loss function and mitigates overfitting, thereby enhancing its efficacy for the classification task of Ransomware Detection.

Logistic Regression (LR): LR is a linear classifier that estimates the probability of a sample belonging to a particular class using the logistic (sigmoid) activation function. It is mainly used in binary classification tasks, using a logistic function (0–1) to estimate probabilities [47]. It is typically trained using maximum likelihood estimation (MLE) by minimizing the cross-entropy (log loss) function.

For the binary classification of ransomware (*y* = 1) versus benign (*y* = 0) based on the fused feature embeddings, the probability function is defined as

(13) $$\hat{P}\left(y=1|x\right)=\sigma \left(w^{T}x+b\right)=\frac{1}{1+e^{-(w^{T}x+b)}}$$

where $\hat{P}$(*y* = 1/*x*) is the predicted probability that *x* belongs to the ransomware class, x is the feature vector, *w* is the weight vector learned during training, *b* is the bias term, $w^{T}x$ is the linear combination of features, and σ (⋅) is the sigmoid activation function.

Logistic Regression provides a simple yet effective baseline classifier to evaluate whether ransomware and benign samples are linearly separable in the feature space. Although more complex models (e.g., RL-guided residual MLP or XGBoost) can capture nonlinear interactions, LR offers interpretability and serves as a benchmark against which more advanced classifiers can be compared.

Artificial Neural Network (ANN): ANN consists of multiple fully connected layers that can learn complex nonlinear mappings from inputs to outputs. Each hidden layer transforms its input through a weighted linear combination, followed by a nonlinear activation function [48].

For the *lth* hidden layer,

(14) $$h^{l}=\sigma (W^{\left(l\right)}h^{\left(l-1\right)}+b^{\left(l\right)})$$

where $h^{l}$ is output (activation vector) of the *lth* hidden layer, $h^{\left(l-1\right)}$ is input vector from the (*l* − *1*) ^*th*^ layer $W^{\left(l\right)}$ is the weight matrix mapping inputs to hidden layer *l*, $b^{\left(l\right)}$ is the bias vector of hidden layer *l,* and σ (⋅) is the nonlinear activation function (e.g., ReLU).

The final output layer applies the softmax activation to produce class probabilities for binary classification (ransomware vs. benign):

(15) $$\hat{y}=softmax(W^{L}h^{\left(L-1\right)}+b^{L})$$

where $\hat{y}$ is predicted probability distribution over the two classes, $W^{L}$ is weight matrix of the final output layer, *b*^(*L*)^ is bias vector of the final layer, and *h*^(*L*−1)^ is the output of the last hidden layer before classification.

The network parameters were optimized via backpropagation to minimize categorical cross-entropy. The ANN captures nonlinear interactions within the fused features, making it suitable for ransomware detection tasks.

Convolutional neural networks (CNN): CNN are designed to capture local spatial patterns in images through convolutional layers, followed by pooling, nonlinear activations, and fully connected layers [8,48]. The convolutional operation is defined as

(16) $$h_{i,j}^{\left(k\right)}=\sigma (\sum_{p,q}X_{i+p,j+q}.K_{p,q}^{\left(k\right)}+b^{\left(k\right)})$$

where *X* is the input image, *K*^(*k*)^ is the convolutional kernel, and *h*^(*k*)^ is the resulting feature map. The final classification was performed using a softmax layer.

(17) $$\hat{y}=softmax(Wh+b)$$

where $\hat{y}$ = predicted probability distribution across the two classes (ransomware, benign);

*W* = weight matrix of the final fully connected layer;

*h* = flattened feature vector after convolution + pooling operations;

*b* = bias vector for the classification layer.

## 4. Experiment and Results

This section presents the experimental evaluation of the proposed TL-RL-FusionNet model on two datasets. Dataset 1 was used as the primary benchmark to compare the proposed RL-guided residual MLP with the baseline classifiers with and without RL-based sample weighting. Dataset 2, the public EldeRan benchmark, was used for external validation using the same TL-RL-FusionNet architecture.

### 4.1. Experimental Setup

The experimental pipeline was implemented in Python 3.10.12 using TensorFlow 2.20.0/Keras 3.13.2, Scikit-learn 1.6.1, XGBoost 3.3.0, NumPy 2.0.2, Pandas 2.2.2, Matplotlib 3.10.0, Seaborn 0.13.2, and psutil 5.9.5. TensorFlow and Keras were used to construct the frozen EfficientNetB0 and InceptionV3 feature extractors and residual MLP classifier. Scikit-learn supports baseline classifiers, stratified cross-validation, preprocessing, and evaluation metrics. XGBoost was used for gradient-boosting-based classification. NumPy and Pandas were used for numerical processing and for handling structured data. Matplotlib and Seaborn were used for result visualization, including training curves, confusion matrices, ROC curves, and interpretability plots, whereas psutil monitored runtime memory consumption.

All experiments were conducted using Google Colab Pro with 51 GB of RAM. The same implementation environment was used for Datasets 1 and 2 to ensure consistent feature transformation, dual-CNN embedding extraction, RL-guided sample weighting, classifier training, performance evaluation, and computational efficiency.

### 4.2. Evaluation Metrics

The model performance was evaluated using accuracy, precision, recall, F1-score, and area under the ROC curve (AUC). These metrics were derived from the confusion matrix, which consists of true positives (TP), true negatives (TN), false positives (FP), and false negatives (FN). In this study, TP and TN denote correctly classified ransomware and benign samples, respectively, while FP denotes benign samples incorrectly classified as ransomware and FN denotes ransomware samples incorrectly classified as benign. Recall is particularly important in ransomware detection because false negatives correspond to undetected ransomware infections, whereas precision reflects the ability to reduce false alarms. The formal definitions of the evaluation metrics are listed in Table 5.

In the context of ransomware detection, these metrics are particularly significant because minimizing false negatives is critical to prevent undetected ransomware infections, whereas reducing false positives helps avoid benign software misclassification, thereby enhancing the practical reliability of the proposed approach under the evaluated experimental setting.

### 4.3. Dataset Splitting and Evaluation Strategy

All experiments were evaluated using a stratified five-fold cross-validation. In each fold, 80% of the data were used for training, and the remaining 20% were held out for testing, whereas the stratification preserved the ransomware–benign ratio across the folds.

The RL agent followed the bandit-style adaptive sample weighting (Section 3.4.1). Within each fold, a fresh Q-table guided a two-stage probe-then-final training procedure on that fold’s training partition only and was discarded afterward. The held-out fold was used only for final performance reporting and was never used to train the classifier or compute any reward signal or Q-table update.

Accuracy, precision, recall, F1-score, and AUC are presented as mean ± standard deviation across five folds. To prevent test-set information from influencing preprocessing, all feature scaling was fitted only on the training partition of each fold and then applied to the corresponding held-out fold. Similarly, the fold-local Q-table was initialized and updated only using the same training partition and was never updated using the held-out test partition. Thus, no test-fold information entered either the preprocessing or RL-guided weighting steps. The fold-level predictions were aggregated to generate confusion matrices and ROC curves.

### 4.4. Dataset 1 Results: Primary Benchmark

This section evaluates the TL-RL-FusionNet on Dataset 1, the behavioral ransomware benchmark. The RL-guided residual MLP was compared with six baseline classifiers, each evaluated with and without RL-based sample weighting, to assess the contribution of adaptive weighting.

The evaluation covered six aspects: cross-validation performance, statistical significance analysis, confusion matrix and ROC analysis, end-to-end efficiency, post hoc interpretability using t-SNE, Grad-CAM mapping, and grid-order sensitivity analysis.

#### 4.4.1. Predictive Performance with and Without RL

Table 6 reports the five-fold cross-validation results for all seven classifiers with and without RL-guided sample weighting. The results show that the RL-guided sample weighting improves every classifier. The proposed residual MLP exhibited the best performance. With RL, it obtained 99.20%, 99.01%, 99.40%, 99.20% F1-score, and 99.84% AUC. Compared with the non-RL residual MLP, the RL configuration improved the accuracy, F1-score, and AUC from 98.70%, 98.71%, and 99.75% to 99.20%, 99.20%, and 99.84%, respectively. The recall increased from 99.20% to 99.40%, which is important for ransomware detection because a higher recall reduces false negatives, where ransomware samples remain undetected.

Moderate and consistent gains were observed for the remaining classifiers. ANN improved from 0.9750 to 0.9810 accuracy, CNN from 0.9750 to 0.9790, RF from 0.9750 to 0.9780, XGBoost from 0.9700 to 0.9760, SVM from 0.9630 to 0.9660, and LR from 0.9180 to 0.9420. These results suggest that RL-guided weighting provides consistent mean performance gains across traditional ML, ensemble, and deep learning classifiers.

SVM also benefited from RL weighting, particularly in recall, which increased from 0.9780 to 0.9840. LR showed the largest absolute improvement, with accuracy increasing by 0.0240 and recall increasing from 0.9080 to 0.9460, indicating that adaptive sample weighting can also be particularly useful for simpler linear models.

Overall, the corrected fold-local RL configuration improved the mean predictive performance of all evaluated classifiers, whereas the residual MLP remained the strongest model on Dataset 1.

To provide uncertainty estimates around the five-fold results, 95% confidence intervals (CI) were computed across the five cross-validation folds using the t-distribution. The confidence interval was calculated as

(18) $${CI}_{95\%}=\bar{x}\pm t_{\left(0.975,n-1\right)}\times \frac{s}{\sqrt{n}}$$

where $\bar{x}$ and *s* are the fold-level mean and standard deviation already reported in Table 6, *n* = 5 is the number of cross-validation folds, and *t*_(0.975,4)_ = 2.776 is the two-sided critical value of the t-distribution at 4 degrees of freedom; the t-distribution (rather than a z-based interval) was used to account for the small number of folds, and any computed upper bound exceeding 1.0 was capped at 1.0 because the reported metrics cannot exceed 100%. The confidence intervals for the proposed residual MLP with and without RL on Dataset 1 are reported in Table 7.

As shown in Table 7, the 95% confidence interval for the RL-guided residual MLP on Dataset 1 ranged from 98.64% to 99.76% for accuracy, 98.15% to 99.87% for precision, 98.29% to 100.00% for recall, 98.64% to 99.76% for F1-score, and 99.57% to 100.00% for AUC. For the non-RL residual MLP, the corresponding intervals were 97.66–99.74% for accuracy, 96.48–100.00% for precision, 97.59–100.00% for recall, 97.68–99.74% for the F1-score, and 99.35–100.00% for the AUC. The confidence intervals show that the MLP configurations achieved a stable fold-level performance. The RL-guided MLP produced higher mean values across all metrics, whereas the intervals overlapped because of the small five-fold sample size. Therefore, the CI analysis supports the conservative interpretation that RL provides consistent positive mean improvement rather than a statistically significant superiority.

#### 4.4.2. Statistical Significance Analysis

To assess whether the corrected RL-guided improvement was consistent across the folds, a paired Wilcoxon signed-rank test was applied to the fold-level results of the proposed residual MLP with and without RL. Table 7 reports the results of this analysis.

As shown in Table 8, the RL configuration outperformed the non-RL configuration in every fold for accuracy (mean difference = +0.0050, W = 0, *p* = 0.125, rank-biserial r = 1.00) and F1-score (mean difference = +0.0049, W = 0, *p* = 0.125, r = 1.00), and also improved recall (mean difference = +0.0020, W = 0, *p* = 0.500, r = 1.00) and AUC (mean difference = +0.0009, W = 1, *p* = 0.500, r = 0.667). These results indicate a consistently positive effect across the cross-validation folds. With only five paired folds, none of the four metrics reached *p* < 0.05; therefore, we do not claim statistically significant superiority. Instead, the Wilcoxon results are interpreted conservatively as consistent positive mean improvements supported by rank-biserial effect sizes and paired-fold direction. The grid-order sensitivity analysis in Section 4.4.6 provides separate evidence of representation robustness.

#### 4.4.3. Confusion Matrix and ROC Analysis

**Figure 4:**
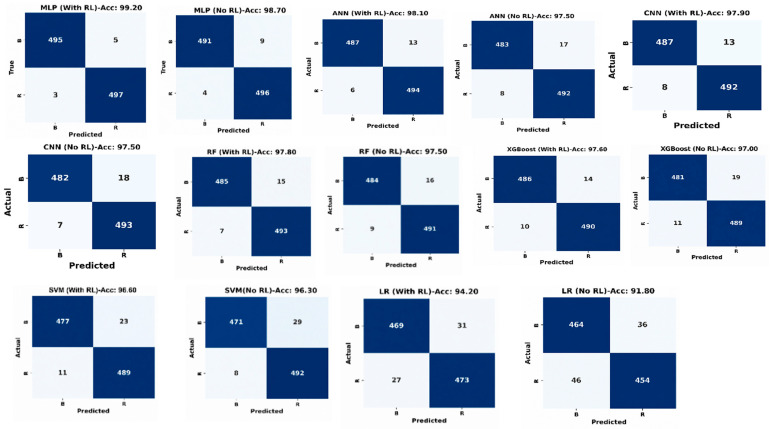
Aggregated confusion matrices for Dataset 1 classifiers with and without RL-guided sample weighting.

**Figure 5:**
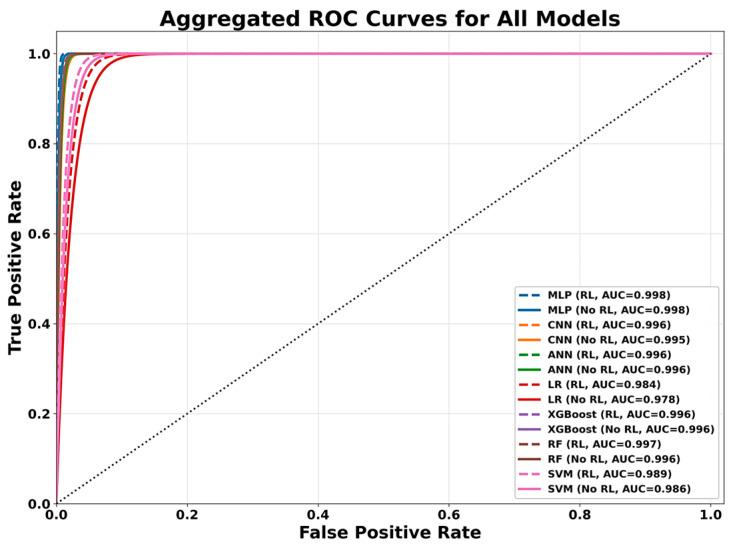
Aggregated ROC curves for all Dataset 1 classifiers using the AUC values from Table 6.

This subsection examines the aggregated confusion matrices and ROC curves for all classifiers with and without RL-guided sample weighting, complementing the summary statistics in Table 6. Figure 4 presents the pooled confusion matrices (fold-aggregated true negatives, false positives, false negatives, and true positives across five-fold cross-validation), and Figure 5 presents the corresponding aggregated ROC curves.

The RL-guided residual MLP produced the fewest errors (5 FP + 3 FN = 8 errors, 99.20% accuracy) compared with 13 errors (9 FP + 4 FN, 98.70%) without RL. The pattern holds across classifiers: RL-guided weighting reduced misclassifications relative to the non-RL configuration for ANN (19 vs. 25 errors), CNN (21 vs. 25 errors), RF (22 vs. 25 errors), and SVM (34 vs. 37 errors) classifiers. The effect was pronounced for logistic regression, where RL reduced errors from 82 (36 FP + 46 FN) to 58 (31 FP + 27 FN)—roughly 29—reducing false negatives from 46 to 27, consistent with the accuracy and recall gains for LR in Table 6. This reduction is relevant for ransomware detection because a false negative corresponds to an undetected ransomware infection.

The aggregated ROC curves (Figure 5) show that all seven classifiers maintain strong threshold-independent discrimination, with AUC values ranging from 0.978 (LR, without RL) to 0.998 (MLP, with and without RL). MLP, CNN, ANN, RF, and XGBoost cluster tightly near-perfect discrimination (AUC ≥ 0.995 in nearly all configurations), while LR and SVM show comparatively greater separation between their RL and non-RL curves—LR improves from AUC 0.978 to 0.984, and SVM improves from 0.986 to 0.989—reinforcing that RL-guided weighting provides the largest discriminative benefit for the weaker linear and margin-based baselines, while offering a smaller but still consistent benefit for the stronger nonlinear classifiers.

To complement the aggregate metrics in Table 6, Table 9 reports the class-wise precision, recall, and F1-score computed from the pooled confusion matrices in Figure 4. The class-wise results show a balanced performance across both classes. With RL, the residual MLP achieved a balanced performance across both classes (Benign: *p* = 99.40%, R = 99.00%, F1 = 99.20%; Ransomware: *p* = 99.00%, R = 99.40%, F1 = 99.20%). Without RL, ransomware recall was slightly lower than benign precision (Benign: *p* = 99.19%, R = 98.20%, F1 = 98.69%; Ransomware: *p* = 98.22%, R = 99.20%, F1 = 98.71%), and RL-guided weighting increased benign recall from 98.20% to 99.00%.

This result indicates that the high aggregate performance is not caused by favoring one class and that the model maintains a very low false-negative behavior for ransomware detection.

#### 4.4.4. End-to-End Computational Efficiency

**Figure 6:**
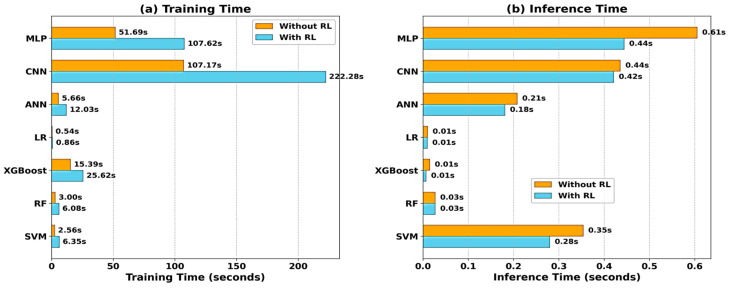
Training and inference times with and without RL. Training time increases moderately under RL, especially for CNN and MLP; inference time remains low and comparable or lower under RL for most models.

**Figure 7:**
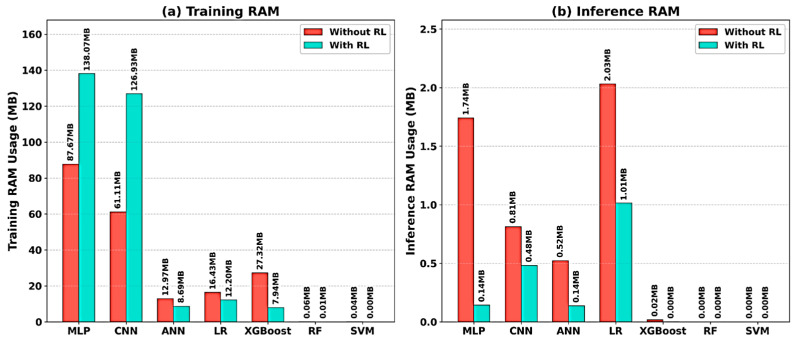
Training and inference RAM with and without RL. The training RAM increases under RL for MLP and CNN but decreases for ANN and XGBoost; the inference RAM is consistently lower under RL for MLP, CNN, ANN, and LR.

To evaluate the computational cost of the proposed framework, we measured the full end-to-end experimental pipeline from raw image loading through frozen dual-CNN feature extraction to classifier training and inference, instead of reporting the classifier training time in isolation. Table 10 provides a component-level breakdown of the proposed residual MLP, and Table 11 extends this comparison to all seven evaluated classifiers with and without RL-guided sample weighting. Figure 6 visualizes the training and inference times, and Figure 7 visualizes the training and inference RAM usage across all models.

The predominant cost in the shared image-to-embedding stage, which involves loading, resizing, and extracting frozen CNN features, is 199.60 s and 730.04 MB. This cost is incurred once, regardless of the classifier used subsequently. Against this baseline, RL-guided training adds a modest, model-dependent overhead from its added probe-training and Q-table update steps, most visible for MLP (107.62 s vs. 51.69 s) and CNN (222.28 s vs. 107.17 s), and minor for lightweight classifiers such as LR, RF, SVM, and XGBoost, whose totals remain dominated by the shared-embedding stage. This overhead is training-only: RL is never invoked at inference and, therefore, it adds no additional inference-time computational cost under the evaluated experimental setting. RL-guided training also tends to reduce inference memory: the MLP inference RAM drops from 1.74 to 0.14 MB and the LR from 2.03 to 1.02 MB, indicating more compact fitted models in addition to the accuracy gains reported in Table 6. Inference time remains low across all models (≤0.61 s) and is generally comparable or better under RL for MLP, ANN, XGBoost, and SVM.

Overall, RL-guided sample weighting improves the detection performance (Table 6) and reduces misclassification (Section 4.4.3) at a training-time cost that is small relative to the shared feature-extraction overhead, while maintaining a low inference overhead under the evaluated experimental setting.

#### 4.4.5. Interpretability Analysis

To verify that the model learns meaningful behavioral patterns rather than spurious correlations, we conducted three interpretability analyses: t-SNE projection of the learned feature space, t-SNE misclassification analysis, and Grad-CAM mapped back to the original behavioral features.

**t-SNE and Misclassification Analysis.**

**Figure 8:**
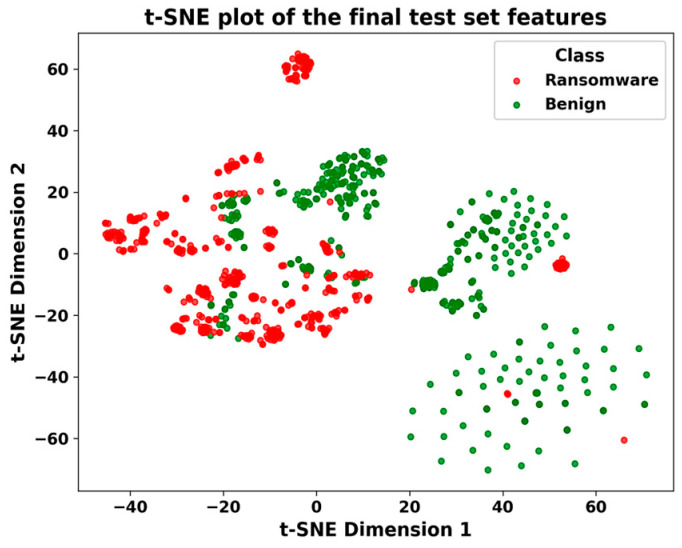
t-SNE projection of test-set features, showing largely separable ransomware (red) and benign (green) clusters with partial central overlap.

**Figure 9:**
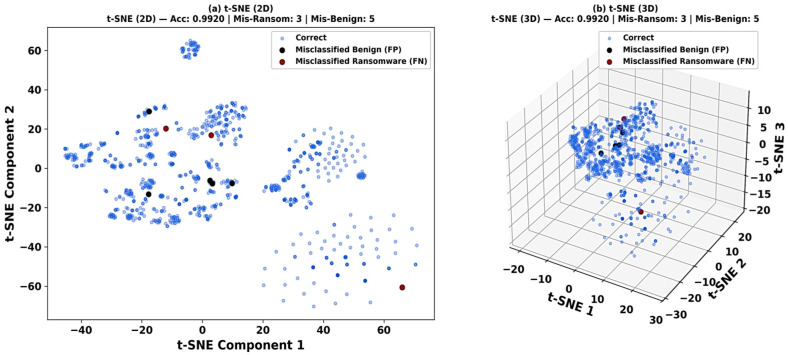
t-SNE misclassification analysis (2D and 3D). Misclassified samples (black: FP, dark red: FN) concentrate near cluster boundaries rather than appearing randomly.

Figure 8 shows that the ransomware and benign samples formed largely separable clusters, with partial overlap in a central low-density region corresponding to behaviorally ambiguous samples. Figure 9 overlays the pooled test-set errors (three false negatives, five false positives; 99.20% accuracy) on this embedding. All eight misclassifications fall at cluster boundaries or within the overlap region rather than being scattered randomly through well-separated clusters, indicating that the model’s errors are systematically tied to behaviorally ambiguous samples rather than arbitrary embedding artifacts, which is consistent with the confusion matrix results in Section 4.4.3.

**Grad-CAM Mapped to Behavioral Features.**

**Figure 10:**
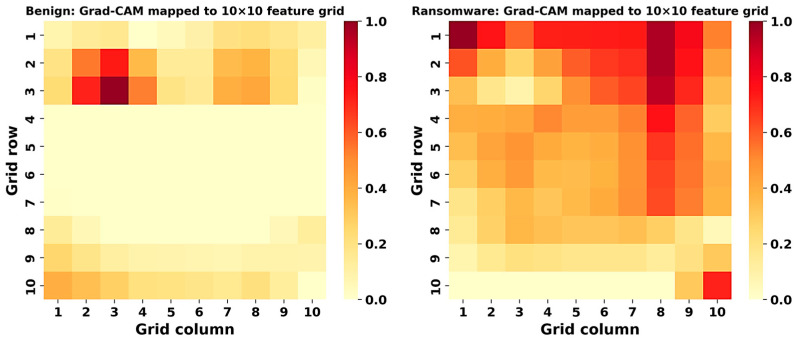
Grad-CAM activation mapped onto the 10 × 10 behavioral grids for a benign sample (left, localized activation) and a ransomware sample (right, broader-distributed activation).

As shown in Figure 10, the Grad-CAM activations were projected onto a 10 × 10 feature grid to identify which behavioral indicators drove each prediction. Activation appears in three distinct regions rather than two: a primary cluster at rows 2–3, columns 2–4 (ranks 1–5, 0.5418–1.0000); a secondary band at rows 2–3, column 7–8 (ranks 7, 8, 10—0.3523–0.3817); and an isolated cell at (10, 1) (rank 9, 0.3754), comparable in strength to the secondary band despite being spatially unrelated to it. The ransomware description (row 1 broadly elevated, column 8 vertical band rows 1–5, corner hotspot at (10, 10)) was consistent with both the table and the heatmap.

The ransomware heatmap shows a markedly different pattern: strong activation spans row 1 across most columns, with additional concentration through rows 2–7 in columns 7–9 and an isolated hot cell at the bottom-right corner. This matches Table 12’s ransomware ranking, where the top ten positions span row 1 (columns 1, 7, 8, 9), rows 2–5 (columns 8–9), and the grid corner (10,10), reflecting the broader, more spatially distributed activation footprint visible in the heatmap, consistent with the model drawing on a wider set of behavioral indicators to identify ransomware than to confirm benign activity.

#### 4.4.6. Grid-Order Sensitivity Analysis

To further strengthen the evaluation and address representation-level robustness, a grid-order sensitivity analysis was performed to examine whether the proposed image-based representation depends strongly on the deterministic 10 × 10 feature-grid arrangement. The proposed framework converts 100 behavioral indicators into a fixed 10 × 10 grid image before CNN feature extraction. Because pretrained CNNs can exploit local spatial patterns, we tested whether the detection performance depends on the specific spatial ordering of features within the grid or only on their presence and value. The RL-guided residual MLP was used for this analysis, consistent with the model configuration reported as the primary result in Section 4.4.

In the canonical configuration, the 100 features occupy fixed grid positions in the deterministic order used in this study. Five additional configurations were generated by randomly permuting 100 grid cell assignments. Each permutation was applied identically to every sample, preserving the feature values while changing the spatial locations. Each shuffled grid was resized to 224 × 224 pixels and passed through the same frozen EfficientNetB0/InceptionV3 extraction, RL-guided sample weighting, and five-fold cross-validation protocol used for the canonical grid. Table 13 reports the per-configuration results, and Table 14 reports the aggregate comparison between the canonical grid and pooled shuffled configurations.

As shown in Table 13, the canonical grid exhibited the highest overall performance. All five random permutations retained high accuracy (0.9840–0.9890), with permutation 2 showing the largest reduction (−0.0080 vs. canonical).

**Figure 11:**
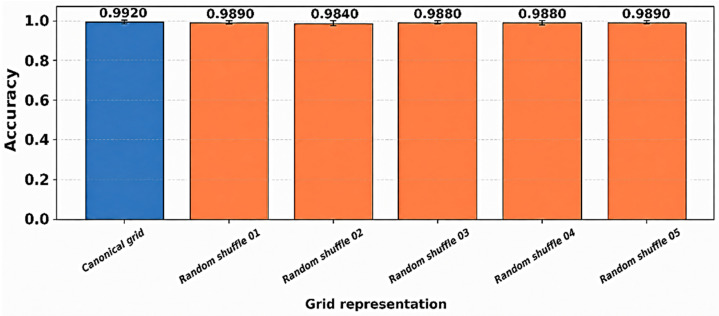
Accuracy of the canonical 10 × 10 grid vs. five randomly permuted grid configurations (RL-guided residual MLP). All permutations retain high accuracy; the canonical layout achieves the best overall performance.

**Figure 12:**
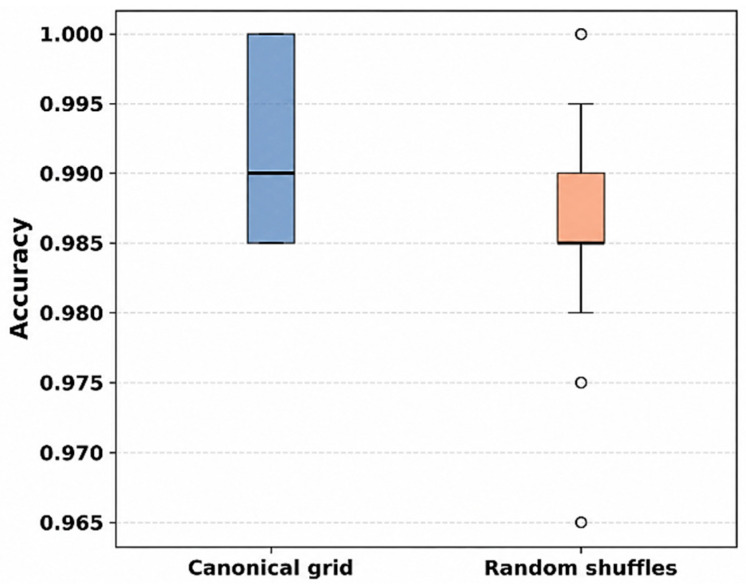
Fold-level accuracy distribution: canonical grid vs. pooled permuted-grid results (RL-guided residual MLP). Permuted layouts show slightly lower median accuracy and greater variability. Boxes show the interquartile range (IQR) with the median line; whiskers extend to 1.5× IQR; circles denote outlier folds.

Table 14 summarizes that the mean accuracy decreased by only 0.0044 compared to the canonical grid across the five permutations (Figure 11). Additionally, fold-level accuracy exhibited a slightly greater variation under permutation than that under the canonical layout (Figure 12). These findings suggest that the classifier performance is not reliant on a specific fixed grid arrangement, as it remains high even with arbitrary spatial reordering of the same 100 features. However, the slight but consistent reduction in accuracy indicates that spatial arrangement is not entirely irrelevant to the CNN-derived embedding. The canonical grid was maintained as the primary representation owing to its reproducibility and marginally superior performance.

### 4.5. Dataset 2 Results: External Validation on EldeRan

This section evaluates TL-RL-FusionNet on EldeRan as an external public benchmark using the same RL protocol applied to Dataset 1 (Section 4.4). Two feature configurations were tested: the complete 30,967-feature representation and the Mutual Information (MI)-selected top-100 subset. Consistent with the scoping in Section 4.4, this evaluation focuses on the proposed MLP with and without RL, serving as external validation rather than a second full baseline benchmark.

#### 4.5.1. EldeRan Predictive Performance

Table 15 presents the corrected fold-local RL results for both feature configurations.

Table 15 demonstrates that the RL enhanced all metrics across both configurations. With the complete feature set, the accuracy increased from 89.59% to 90.36%, and the recall improved from 88.98% to 90.18%. The MI top-100 configuration exhibited the highest overall performance, achieving 92.08% accuracy, 93.46% recall, 92.18% F1-score, and 98.13% AUC with RL compared to 91.39%, 91.91%, 91.38%, and 97.81%, respectively, without RL. This indicates that compact feature selection not only maintains but also slightly surpasses the detection capability of the full feature space under the RL-guided protocol.

Using the same t-distribution-based procedure described in Section 4.4.1, 95% confidence intervals were computed for the EldeRan results across the five cross-validation folds. The same setting was used, with *n* = 5, *df* = 4, and *t*_0.975_,_4_ = 2.776. The resulting confidence intervals for the full EldeRan feature representation and MI-selected top-100 representation are reported in Table 16.

As shown in Table 16, for the RL-guided residual MLP on the EldeRan_ALL configuration, the 95% confidence interval ranged from 88.81% to 91.91% for accuracy, from 88.48% to 92.60% for precision, from 86.64% to 93.72% for recall, from 88.64% to 92.02% for the F1-score, and from 95.72% to 96.94% for the AUC. Without RL, the corresponding intervals were 87.21–91.97% (accuracy), 87.88–92.30% (precision), 84.52–93.44% (recall), 86.89–92.11% (F1-score), and 95.32–97.10% (AUC). For the EldeRan_MI100 configuration, the RL-guided intervals ranged from 89.37% to 94.79% for accuracy, 89.04% to 92.98% for precision, 89.41% to 97.51% for recall, 89.47% to 94.89% for F1-score, and 97.15% to 99.11% for AUC, whereas the non-RL intervals were 88.40–94.38% (accuracy), 88.13–93.85% (precision), 85.28–98.54% (recall), 88.10–94.66% (F1-score), and 96.90–98.72% (AUC). Across both configurations, the RL-guided models achieved consistently higher mean values than the corresponding non-RL models, while the confidence intervals overlapped across several metrics because of the small five-fold sample size. The MI-selected top-100 representation also achieved stronger mean performance than the full feature representation, suggesting that compact discriminative features were more effective than the complete high-dimensional EldeRan feature space. Therefore, the CI analysis supports the conservative interpretation that RL provides consistent positive mean improvement rather than statistically significant superiority.

#### 4.5.2. Statistical Significance of the RL Improvement (EldeRan)

To assess whether RL-guided improvement on the external EldeRan benchmark reflects a consistent effect, a paired Wilcoxon signed-rank test was applied to five fold-level pairs (with RL vs. without RL) for MI top-100 configuration. Table 17 reports the results.

As reported in Table 17, in the EldeRan MI top-100 configuration, the RL-guided model outperformed the non-RL model in every fold for accuracy (mean difference = +0.0069, W = 1.0, *p* = 0.125, rank-biserial r = 1.00), F1-score (mean difference = +0.0080, W = 1.0, *p* = 0.250, r = 1.00), and recall (mean difference = +0.0155, W = 0.0, *p* = 0.250, r = 1.00), and in most folds for AUC (mean difference = +0.0032, W = 1.0, *p* = 0.125, r = 0.867). Similar to Dataset 1 (Section 4.4.2), the small paired sample size of five folds limits statistical power; therefore, we do not claim statistically significant superiority at *p* < 0.05. Instead, the Wilcoxon results were interpreted as consistent positive mean improvements supported by strong rank-biserial effect sizes and paired-fold direction. These results reinforce the pattern observed in Dataset 1 and suggest that the RL-guided weighting effect remains directionally stable on the external EldeRan benchmark.

#### 4.5.3. Confusion Matrix and ROC Analysis

**Figure 13:**
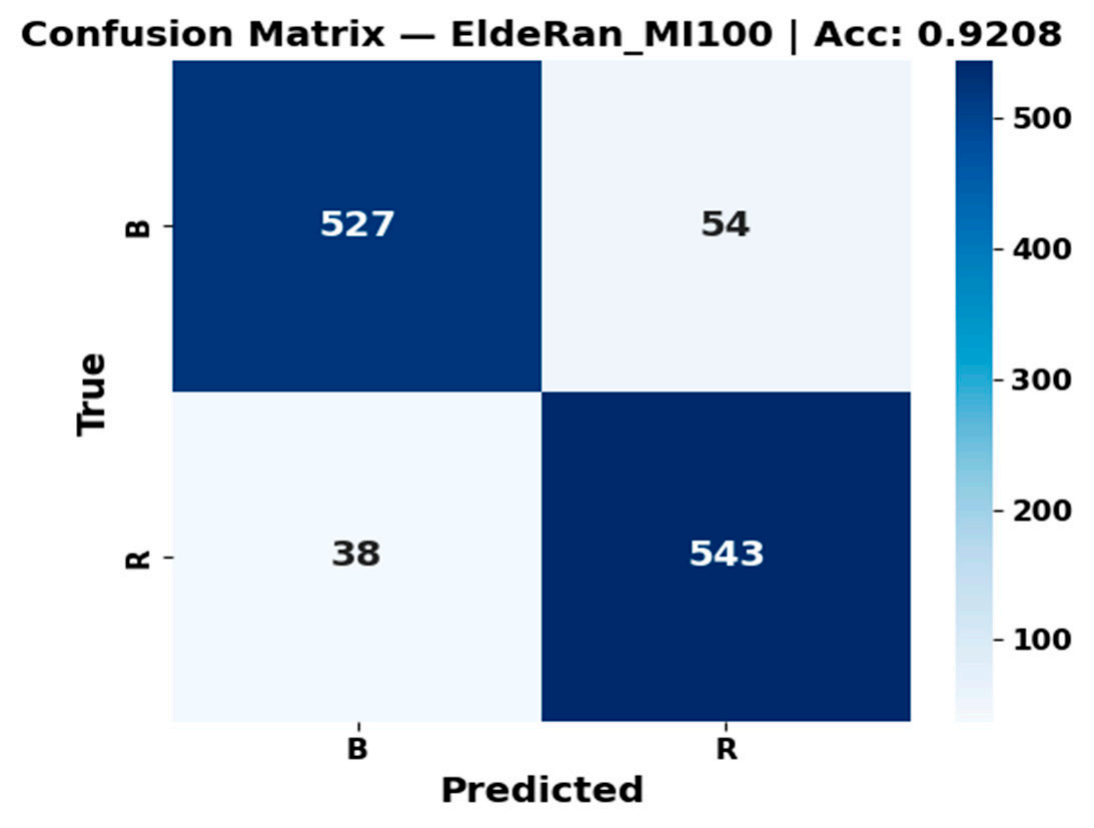
Confusion matrix for EldeRan MI top-100 under fold-local RL (pooled, five folds). 527 benign and 543 ransomware samples correctly classified; 54 FP, 38 FN; accuracy = 92.08%.

**Figure 14:**
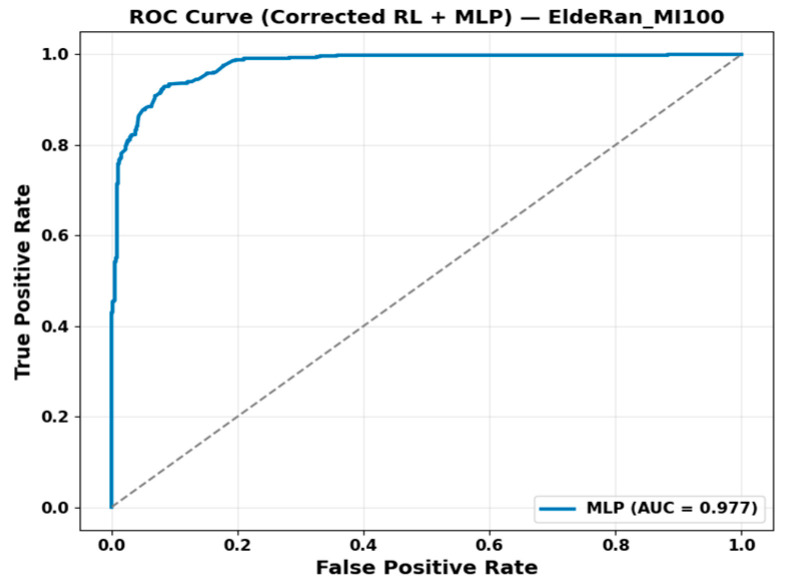
ROC curve for EldeRan MI top-100 under RL-guided protocol (pooled out-of-fold predictions, AUC = 0.977).

Figure 13 and Figure 14 show the pooled confusion matrix and ROC curve for the best-performing configuration (MI top-100 with RL). The model correctly classified 527 benign and 543 ransomware samples, with 54 and 38 false positives and false negatives, respectively (92.08% accuracy), which is consistent with the mean fold accuracy in Table 15. The higher false-positive count relative to false negatives reflects a mild bias towards flagging benign samples as ransomware—the operationally safer error direction, because missed detections carry a greater risk than false alarms. The pooled ROC curve shows an AUC of 0.977, consistent with the mean per-fold AUC of 0.9813 ± 0.0079 in Table 15, confirming a strong threshold-independent separability for this external benchmark.

Table 18 reports the class-wise precision, recall, and F1-score computed from the pooled confusion matrices in Figure 13. The class-wise results show broadly balanced performance across both classes, with a mild shift toward higher ransomware sensitivity. With RL, the residual MLP achieved strong performance for both classes: Benign: *p* = 93.27%, R = 90.71%, F1 = 91.97%; Ransomware: *p* = 90.95%, R = 93.46%, F1 = 92.19%. Without RL, the corresponding results were Benign: *p* = 91.83%, R = 90.88%, F1 = 91.35%; Ransomware: *p* = 90.99%, R = 91.91%, F1 = 91.38%. RL-guided weighting increased ransomware recall from 91.91% to 93.46%, which directly reduces the number of ransomware samples misclassified as benign.

These results indicate that RL-guided weighting improves both classes jointly rather than introducing a trade-off between benign and ransomware performance. As with Dataset 1, the largest gain occurred in ransomware recall, which is operationally important because a missed ransomware infection, or false negative, is typically more costly than a false alarm on benign software.

#### 4.5.4. EldeRan Computational Efficiency

Table 19 reports the end-to-end computational cost of the EldeRan pipeline for both feature configurations, with and without the RL-guided sample weighting.

Similar to Dataset 1 (Section 4.4.4), loading and preprocessing remained consistent across the RL settings for a feature set, with a duration of 33.448 s for both, since preprocessing occurred prior to feature selection. However, the preprocessing RAM usage varied with dimensionality, with 687.85 MB required for the 30,967-feature representation and 667.25 MB for the MI top-100 subset. RL-guided training increased the mean training time compared with the non-RL configuration for both feature sets: from 55.88 s to 111.53 s for EldeRan_ALL and from 56.06 s to 109.08 s for EldeRan_MI100. This increase aligns with the probe-training and Q-table update steps noted for Dataset 1. This overhead is limited to the training phase because RL is not utilized during inference.

RL-guided training reduces inference-time RAM usage versus non-RL across feature settings—from 3.497 to 1.397 MB for the full feature set and from 1.422 to 0.621 MB for MI top-100—mirroring compact fitted representations observed for Dataset 1 classifiers in Section 4.4.4. The inference time remained (≤0.44 s) across the configurations, regardless of the RL setting.

#### 4.5.5. EldeRan Interpretability Analysis

We applied the same t-SNE, misclassification, and Grad-CAM analyses used for Dataset 1 (Section 4.4.5) to the EldeRan MI top-100 configuration.

**t-SNE and Misclassification**

**Figure 15:**
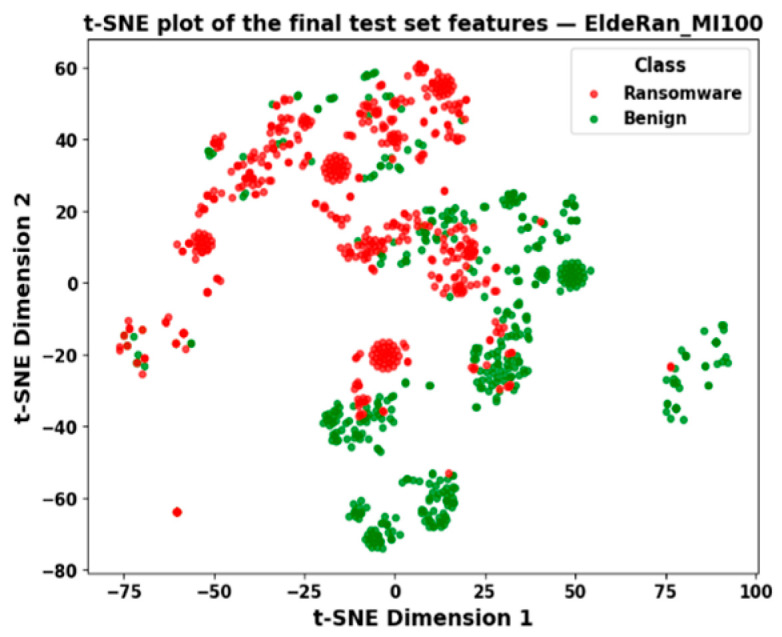
t-SNE projection, EldeRan MI top-100: partially separable clusters with greater overlap than Dataset 1.

**Figure 16:**
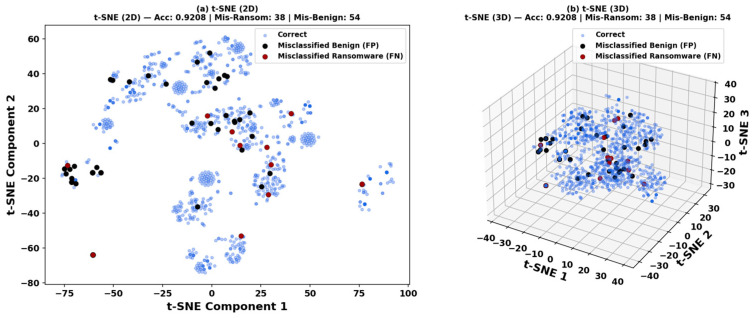
Misclassification analysis (2D/3D t-SNE), EldeRan MI top-100: errors concentrated near cluster boundaries.

Figure 15 shows ransomware (red) and benign (green) samples forming partially separable clusters, with more overlap than Dataset 1 (Figure 8), which is consistent with EldeRan’s lower accuracy (92.08% vs. 99.20%). Figure 16 overlays the pooled misclassifications (38 FN, 54 FP) on the embedding. As with Dataset 1, the errors were concentrated near the cluster boundaries and overlap regions rather than appearing randomly, indicating that the model errors tracked behaviorally ambiguous samples.

**Grad-CAM on MI100 Grid**

**Figure 17:**
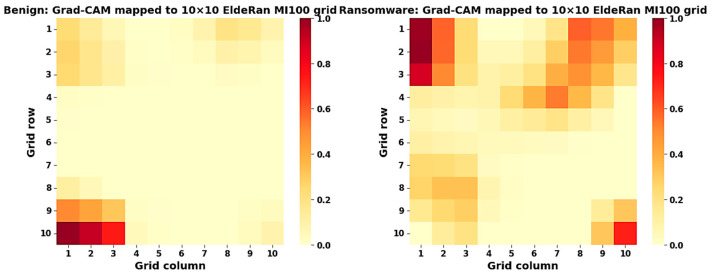
Grad-CAM activation on the 10 × 10 MI top-100 grid: benign (concentrated in the bottom-left block with a fainter secondary trace) versus ransomware (distributed across three regions, including an isolated corner cell).

As shown in Figure 17, the EldeRan benign heatmap shows activation concentrated almost entirely in the bottom-left region (rows 9 and 10, columns 1–3) with a much fainter secondary trace near rows 1–3 and column 1. This matches Table 20’s benign ranking directly: the top six positions—(10, 1), (10, 2), (10, 3), (9, 1), (9, 2), (9, 3)—occupy exactly that bottom-left block (activations 0.3156–1.0000), while ranks 7–9—(2, 1), (3, 1), (1, 1)—correspond to the fainter secondary trace in column 1 (0.2359–0.2652).

For the ransomware heatmap, activation appears in three regions: a primary top-left cluster at rows 1–3, columns 1–2; a secondary cluster at rows 1–2, columns 8–9; and two isolated points at the corner cell (10, 10) and position (4, 7). This pattern is consistent with Table 15, where the top ransomware-ranked positions include (2, 1), (1, 1), (3, 1), (10, 10), (1, 8), (1, 2), (2, 2), (1, 9), (2, 8), and (4, 7). The benign heatmap remains dominated by the bottom-left region and a weaker column-1 trace.

### 4.6. Comparison with Existing Relevant Methods

The performance of TL-RL-FusionNet was compared with that of related ransomware detection studies using pretrained CNN feature extractors, standalone classifiers, or reinforcement-learning-based strategies (Table 21). Because the listed studies differ in dataset composition, feature engineering, execution environment, and family coverage, the conclusions below are drawn from reported numerical metrics under each study’s own conditions, not from a static/dynamic category.

The comparison shows that TL-RL-FusionNet achieves competitive performance relative to prior ransomware detection approaches under its evaluation conditions. On Dataset 1, TL-RL-FusionNet’s 99.20% accuracy exceeds the highest reported baseline (99.00%, [14]) by 0.20%, and it is the only method in the table to report a complete metric set: recall-relevant FP/FN rates, inference memory, and both training and prediction times. The prediction time (0.444 s) was faster than that of [12] (0.471 s) and substantially faster than that of [20] (0.76 s). The training time (107.62 s) was higher than that in [14] (18.928 s) but remained well below that in [20] (168.27 s). The added training cost reflects the fold-local RL-guided sample-weighting mechanism (Section 4.4.4) and is not incurred at inference, where the model memory footprint (0.143 MB) and prediction time remain low. No baseline in the table reports FP/FN rates; therefore, TL-RL-FusionNet’s 1.00% FP and 0.60% FN cannot be directly compared, but they quantify the low error rates achieved on both classes.

Compared with [20], the only other RL-based ransomware detection method with reported accuracy, TL-RL-FusionNet’s Dataset 1 result is 9.42 points higher (99.20% vs. 89.78%) with a faster prediction time despite its higher training cost. Compared to [31] (>98% accuracy, federated RL for IoT malware mitigation), no meaningful numerical comparison applies: it addresses a different task (decentralized malware mitigation) and does not report recall, FN rate, prediction time, or inference memory.

On EldeRan MI top-100, TL-RL-FusionNet achieved 92.08% accuracy with 0.351 s prediction time and 0.621 MB inference memory. No listed study reported EldeRan results under a comparable protocol; therefore, this serves to demonstrate the generalization to an independent public dataset rather than further comparison. Its higher FP (9.29%) and FN (6.54%) rates relative to Dataset 1 reflect the comparatively lower separability of EldeRan’s legacy feature space, which is consistent with the interpretability findings in Section 4.5.5.

TL-RL-FusionNet differs from the compared studies in three respects, as evidenced by the metrics above: it reports a more complete metric set than the compared baselines, most of which report accuracy alone; it applies RL-guided sample weighting during training—a mechanism otherwise reported only in [20,32] but not evaluated for image-based ransomware classification in either—without inference-time overhead; and it evaluates the same architecture on both a custom dataset and the public EldeRan benchmark. These results support the combination of behavioral-to-image transformation, frozen dual-CNN fusion, and RL-guided adaptive weighting for efficient ransomware detection.

## 5. Discussion

This study demonstrates that TL-RL-FusionNet provides an effective ransomware detection framework by integrating behavioral feature-grid transformation, frozen dual CNN embedding extraction, residual MLP classification, and a fold-local bandit-style weighting mechanism. The sample-weighting module is applied only during training: the Q-table is initialized independently in each cross-validation fold and updated only using the corresponding training partition, ensuring a leakage-safe model evaluation.

The results indicate that RL-guided weighting is particularly useful for reducing high-risk detection errors. On Dataset 1, RL-guided weighting improved every evaluated classifier (Table 6), with the proposed residual MLP performing the best while maintaining low inference latency and memory usage. A paired Wilcoxon test (Section 4.4.2, Table 8) showed a consistent direction of improvement across folds, although the small paired sample limited statistical power. The confusion matrix results show that false negatives remained very low, which is important because undetected ransomware represents the most damaging error in operational environments. The t-SNE and Grad-CAM analyses (Section 4.4.5) further showed that errors were concentrated near ambiguous cluster boundaries and that the model’s attention aligned with recognizable behavioral feature-grid patterns. Grid-order sensitivity analysis (Section 4.4.6) showed only a modest 0.44-point accuracy reduction under random feature permutation, indicating that predictions are not an artifact of one fixed layout.

The EldeRan benchmark supports this interpretation from the perspective of an external public dataset. The TL-RL-FusionNet remained effective without architectural modifications. Specifically, the RL demonstrated enhanced performance across both feature configurations. Furthermore, the MI top-100 configuration surpassed the full feature set, achieving an accuracy of 92.08% compared to 90.36% (Table 15). This suggests that compact and discriminative behavioral features can be more useful than large feature spaces with redundant or sparse attributes. Additionally, the RL advantage was consistently confirmed by the Wilcoxon test (Section 4.5.2). EldeRan’s lower overall accuracy and higher error rates relative to Dataset 1 (Table 21) reflect greater class overlap in its legacy feature space rather than a failure of the RL mechanism (Section 4.5.5). We interpret EldeRan as an external validation of architectural portability, not a direct cross-dataset transfer, because the two datasets differ in feature design and execution environment.

The proposed fold-local bandit-style weighting is conceptually related to several established strategies for handling sample difficulty and class imbalance but differs in terms of mechanism and scope. Focal loss down-weights easy samples using a fixed confidence-based modulating factor, whereas class weighting assigns the same weight to all samples in a class and mainly addresses class imbalance. Hard example mining emphasizes high-loss samples but can overfit noisy or ambiguous cases, and curriculum learning requires a predefined difficulty measure and pacing schedule. In contrast, the proposed tabular Q-learning agent treats weighting as a per-sample bandit problem: each training sample receives one of five discrete weights updated using reward feedback from the fold-local probe model rather than a fixed heuristic, class prior, or predefined curriculum. This allows the sample contribution to adapt to the fold-local classifier behavior while remaining computationally lightweight and fully separated from the frozen CNN feature extraction. A direct empirical comparison with focal loss, class weighting, hard-example mining, and curriculum-learning baselines under the same fold-local protocol is left for future studies.

Compared with prior ransomware detection studies, TL-RL-FusionNet offers a practical balance between accuracy, efficiency, and interpretability of the results. Unlike approaches based mainly on PE-derived static attributes or binary image representations [10,23,24], it uses sandbox-derived behavioral evidence to capture runtime files, registries, processes, and network activities. This is important because modern ransomware often employs polymorphism, obfuscation, staged execution, and evasive behaviors, which can reduce the reliability of purely static representations. Existing transfer learning pipelines usually pair pretrained CNN feature extractors with fixed classifiers, such as MLP, SVM, XGBoost, or KNN [12,13,14,15]. In contrast, TL-RL-FusionNet adds fold-local RL-guided sample weighting to make the training adaptive. Although RL has been explored in cybersecurity using PE headers, Android attributes, dynamic embeddings, and federated adaptive defense [18,19,20,32], these studies do not combine frozen dual-CNN feature fusion with RL-guided weighting for behavioral feature-grid ransomware detection. Because RL is used only during training, it does not add any inference-time computational overhead.

This study has several limitations. First, the primary dataset was balanced and moderate in size; therefore, a broader evaluation is required across larger sample collections, additional ransomware families, benign software categories and execution environments. Second, the evaluation uses stratified cross-validation and does not include family-disjoint, temporal, or unseen family ransomware validation. Although the results demonstrate a strong performance under controlled fold-based evaluation, future studies should examine whether the framework generalizes to unseen ransomware families, temporally separated samples, and evolving attack campaigns. Third, although a grid-order sensitivity analysis was conducted using five randomly permuted (10 × 10) layouts, a full structured feature-to-image ablation against tabular representations, alternative image encodings, and different feature-grouping strategies was not performed. Future studies should systematically evaluate how different representation designs affect CNN-derived embeddings and downstream ransomware detection performance. Fourth, although the RL-guided weighting improves adaptivity, it does not constitute a complete adversarial defense; evasive or poisoned samples may still influence the decision boundary. Finally, although EldeRan was used as an external public benchmark, stronger baseline repetition and direct cross-dataset training/testing remain future work because Datasets 1 and EldeRan differ in feature schema, sandbox environment, operating system context, dimensionality, and sample composition.

Overall, fold-local RL-guided sample weighting is the mechanism that makes TL-RL-FusionNet adaptive without compromising evaluation validity, achieving strong performance on both benchmarks while remaining computationally efficient and behaviorally interpretable.

## 6. Conclusions and Future Work

This study presents TL-RL-FusionNet, an adaptive ransomware detection framework combining behavioral feature-to-image transformation, frozen EfficientNetB0/InceptionV3 feature fusion, a residual MLP classifier, and fold-local RL-guided sample weighting, with the RL agent reinitialized independently per fold to ensure a fair with/without RL comparison. On Dataset 1, TL-RL-FusionNet achieved the best overall performance among the evaluated classifiers (99.20% accuracy, 99.01% precision, 99.40% recall, 99.20% F1-score, 99.84% AUC), with consistent RL-driven improvement confirmed by paired statistical testing, meaningful behaviorally grounded representations (t-SNE/Grad-CAM), and only modest sensitivity to feature-grid ordering. On the independent EldeRan benchmark, the same architecture remained effective without modification (90.36% accuracy, full features; 92.08%, MI top-100), with RL improving both; its lower accuracy reflects EldeRan’s lower feature separability rather than a method limitation. The framework avoids costly CNN backbone fine-tuning, and the RL-guided weighting mechanism is used only during the training phase. Therefore, the final classifier did not introduce additional RL-related inference overheads in the evaluated experimental settings.

Future work will pursue (1) family-disjoint or unseen-family evaluation, (2) adversarial robustness testing, (3) larger and more diverse datasets, (4) repeated or nested cross-validation for greater statistical power, (5) cross-dataset training/testing where feature-schema alignment permits, and (6) direct empirical comparison of the proposed fold-local bandit-style sample weighting against focal loss, static class weighting, hard-example mining, and curriculum-learning baselines under an identical stratified cross-validation protocol. TL-RL-FusionNet demonstrates how fold-local reinforcement learning can make transfer-learning-based ransomware detection more adaptive and interpretable while identifying concrete directions for broader validation.

## Figures and Tables

**Table 1:** Comparative summary of related studies on ransomware detection. This table synthesizes prior approaches across ML, DL, TL, and RL, highlighting feature extractors, feature types and analysis techniques, classifiers, datasets, performance, strengths and limitations.

Ref	Feature	Model	Dataset	Acc (%)	Strength	Limitation
**Machine Learning (ML) and Deep Learning (DL)**						
[23]	PE header bytes	LSTM	R = 1000, B = 1000	93.25	Simple DL pipeline without extensive manual feature engineering	No unseen-family evaluation
[24]	PE-header image representation	CNN	R = 1000, B = 1000	93.33	Converts static executable structure into image form	Limited evaluation on unseen ransomware families
[10]	PE header color images	Xception CNN	R = 2023, B = 2134	98.20	Improved static image accuracy	Static features may be affected by packing and obfuscation
[3]	Dynamic features	RLR	R = 582, B = 942	96.3	Simple ML on runtime features	Legacy XP/Cuckoo telemetry
[25].	API-call sequences	TextCNN	R = 1000, B = 1000	95.9	Effective sequence-based behavioural modelling	Lacks interpretability
[26]	API calls, DLLs, mutexes	CNN + XAI	R = 668, B = 668	99.4	Combines dynamic behaviour with explanation methods	Computationally intensive
[27]	Hybrid features	HNN	R≈10.3 K, B≈10 K	98.75	Robust cross-platform evaluation	Higher computational overhead
**Transfer Learning (TL)**						
[28]	APK image representation	19 pretrained CNNs	R = 500, B = 500	99.5	Extensive pretrained-CNN evaluation	May struggle with evolving ransomware variants
[29]	Malware byteplot images	ResNet-50	M = 9339	98.62	Strong TL performance on grayscale byteplots	No detailed imbalance analysis
[30]	Hybrid static and dynamic images	VGG16	M = 512, B = 888	94.70	Combines static and dynamic visual evidence	Limited scalability analysis
[17]	Image-based dynamic features	ResNet50, EfficientNetB0, InceptionV3, Xception,	R = 500, B = 500	99.96	High detection performance	Fine-tuning increases training time and memory cost
[13]	Grayscale images	DenseNet-121, XGBoost	Malimg	98.58	Efficient TL-based feature extraction	No unseen or obfuscated-sample evaluation
[14]	Malware binary images	VGG16, ResNet50, Bi-SVC	Malimg	100	Bimodal stacked features	Single-dataset overfitting risk
[15]	Byteplot grayscale images	VGG16, SVM	M= 10,136	92.97	Reduces manual feature engineering	Lower performance than recent ensemble methods
[12]	Malware binary images	VGG16, VGG19, ResNet50, InceptionV3, MLP	Malimg	98.55	Multi-CNN TL improves robustness to packing	Computationally heavy
[11]	Malware binary images	VGG16, ResNet50, InceptionV3, SVM	Malimg and IoT-Android dataset	98.82	Robustness to obfuscation and polymorphism	Small and older datasets; image-size and imbalance effects not fully studied
**Reinforcement Learning (RL)**						
[18]	Static PE-header features	Double DQN	R = 688	97.9	Early detection without execution risk	Vulnerable to packing
[19]	Dynamic embeddings	RL + Bayesian networks	Vulnerability and system logs	N/A	Adaptive resilience modelling	Not designed for direct ransomware classification
[20]	Android permissions and network traffic	A2C, DQN, DDQN, PPO	Android ransomware dataset	89.78	Improves adaptive decision-making	Limited to static attributes
[31]	Device activity patterns	Federated learning + RL	IoT malware setting	98	Improves adaptability and distributed learning	Scalability underexplored

**Table 2:** Ransomware families (25) included in this study with sample counts (500). “First Seen” denotes the year of the earliest documented public report for each family based on multi-source threat intelligence. Families were selected based on global prevalence, documented impact, and multivendor confirmation of their classification.

Family Name	First Seen	Samples	Family Name	First Seen	Samples	Family Name	First Seen	Samples
LockBit	2019	30	DarkSide	2020	24	MountLocker	2020	15
BlackCat	2021	30	NetWalker	2019	24	Mallox	2021	15
GandCrab	2018	28	Phobos	2018	24	Makop	2020	12
Thanos	2020	28	RagnarLocker	2019	23	Nefilim	2020	11
BlackMatter	2021	28	Conti	2020	18	Clop	2019	10
MedusaLocker	2019	28	Mespinoza/Pysa	2019	18	Cerber	2016	9
Babuk	2021	27	Avaddon	2020	16	Maze	2019	8
REvil/Sodinokibi	2019	26	Ryuk	2018	15	BlueSky	2022	8
WastedLocker	2020	25						

Total ransomware families = 25. Total ransomware samples = 500.

**Table 3:** Residual MLP architectural parameters.

Layer	Output Shape	Parameters
Input	(None, 3328)	0
Dense	(None, 1024)	3,408,896
BatchNormalization	(None, 1024)	4096
Dropout (0.3)	(None, 1024)	0
Residual Block ×2	(None, 1024)	1,053,184
Dense (256)	(None, 256)	262,400
BatchNormalization	(None, 256)	1024
Dense (1024)	(None, 1024)	263,168
Add + ReLU	(None, 1024)	0
Dropout (0.2)	(None, 1024)	0
Output Dense (Softmax, 2)	(None, 2)	2050
Total Parameters		4,468,226

**Table 4:** Training and hyperparameters settings.

Parameter	Value
Input	3328-dimensional fused EfficientNetB0–InceptionV3 embedding
Architecture	Dense (1024) followed by two residual blocks
Residual block	Dense (256, ReLU) → BatchNorm → Dense (1024) → Add + ReLU → Dropout(0.2)
Output	Dense (2) with Softmax activation
Regularization	Dropout 0.3 after initial dense layer; Dropout 0.2 after each residual block
Normalization	BatchNorm after the initial dense layer and after the 256-unit dense layer in each residual block
Optimizer	Adam
Learning Rate	Cosine annealing, initial learning rate 1 × 10^−3^
Loss	Categorical cross-entropy with label smoothing =0.1
Training setup	Batch size = 32; Epochs = 25; Stratified 5-fold CV
RL weighting	Fold-local bandit-style sample weighting

**Table 5:** Evaluation metrics used in this study.

Metric Name	Description	Related Syntax/Equation
Accuracy	The ratio of correctly predicted instances (TP and TN) to the total number of observations.	$Accuracy=\frac{TP+TN}{TP+TN+FP+FN}$
Precision	The ratio of correctly predicted positive observations (TP) to the total predicted positives (TP + FP).	Precision $=\frac{TP}{TP+FP}$
Recall (Sensitivity)	The proportion of actual positives (TP) correctly identified by the model.	Recall $=\frac{TP}{TP+FN}$
F1-Score	The harmonic mean of precision and recall balances the false positives and false negatives.	F1-measure = $2\times \frac{Precision\times recall}{Precision+recall}$
AUC (Area Under ROC Curve)	The classifier performance was evaluated across the thresholds, with higher values indicating stronger separability.	Derived from the ROC curve (TPR vs. FPR).

**Table 6:** Cross-validation performance of Dataset 1 classifiers with and without RL-guided sample weighting, reported as mean ± standard deviation across five-folds.

Model	Configuration	Accuracy	Precision	Recall	F1-Score	AUC
MLP	With RL	0.9920 ± 0.0045	0.9901 ± 0.0069	0.9940 ± 0.0089	0.9920 ± 0.0045	0.9984 ± 0.0022
	Without RL	0.9870 ± 0.0084	0.9824 ± 0.0142	0.9920 ± 0.0130	0.9871 ± 0.0083	0.9975 ± 0.0032
ANN	With RL	0.9810 ± 0.0082	0.9747 ± 0.0173	0.9880 ± 0.0110	0.9812 ± 0.0081	0.9961 ± 0.0036
	Without RL	0.9750 ± 0.0050	0.9669 ± 0.0153	0.9840 ± 0.0114	0.9752 ± 0.0048	0.9955 ± 0.0039
CNN	With RL	0.9790 ± 0.0065	0.9745 ± 0.0141	0.9860 ± 0.0114	0.9791 ± 0.0066	0.9964 ± 0.0027
	Without RL	0.9750 ± 0.0106	0.9651 ± 0.0179	0.9840 ± 0.0167	0.9753 ± 0.0103	0.9950 ± 0.0073
RF	With RL	0.9780 ± 0.0076	0.9705 ± 0.0097	0.9860 ± 0.0089	0.9782 ± 0.0075	0.9965 ± 0.0040
	Without RL	0.9750 ± 0.0079	0.9686 ± 0.0124	0.9820 ± 0.0110	0.9752 ± 0.0078	0.9960 ± 0.0034
XGBoost	With RL	0.9760 ± 0.0089	0.9723 ± 0.0105	0.9800 ± 0.0141	0.9761 ± 0.0090	0.9964 ± 0.0034
	Without RL	0.9700 ± 0.0071	0.9627 ± 0.0124	0.9780 ± 0.0084	0.9703 ± 0.0069	0.9961 ± 0.0037
SVM	With RL	0.9660 ± 0.0055	0.9556 ± 0.0176	0.9840 ± 0.0182	0.9664 ± 0.0054	0.9891 ± 0.0097
	Without RL	0.9630 ± 0.0115	0.9453 ± 0.0284	0.9780 ± 0.0192	0.9639 ± 0.0105	0.9861 ± 0.0089
LR	With RL	0.9420 ± 0.0160	0.9388 ± 0.0166	0.9460 ± 0.0305	0.9421 ± 0.0166	0.9836 ± 0.0078
	Without RL	0.9180 ± 0.0368	0.9264 ± 0.0193	0.9080 ± 0.0680	0.9162 ± 0.0411	0.9780 ± 0.0098

**Table 7:** The 95% confidence intervals (t-distribution, df = 4) for the RL-guided and non-RL residual MLP on Dataset 1, computed from the fold-level mean and standard deviation reported in Table 6.

Model	Configuration	Metric	Mean	Std	95% CI (t, df = 4)
MLP	With RL	Accuracy	0.9920	0.0045	[0.9864, 0.9976]
		Precision	0.9901	0.0069	[0.9815, 0.9987]
		Recall	0.9940	0.0089	[0.9829, 1.0000]
		F1-score	0.9920	0.0045	[0.9864, 0.9976]
		AUC	0.9984	0.0022	[0.9957, 1.0000]
MLP	Without RL	Accuracy	0.9870	0.0084	[0.9766, 0.9974]
		Precision	0.9824	0.0142	[0.9648, 1.0000]
		Recall	0.9920	0.0130	[0.9759, 1.0000]
		F1-score	0.9871	0.0083	[0.9768, 0.9974]
		AUC	0.9975	0.0032	[0.9935, 1.0000]

**Table 8:** Paired Wilcoxon signed-rank test comparing RL and non-RL residual MLP on Dataset 1 (*n* = 5 paired folds).

Metric	With RL Mean	Non-RL Mean	Mean Difference (RL − Non-RL)	Wilcoxon W	*p*-Value	Rank-Biserial r	Direction
Accuracy	0.9920	0.9870	0.0050	0	0.125	1	RL higher
Recall	0.9940	0.9920	0.0020	0	0.5	1	RL higher
F1 Score	0.9920	0.9871	0.0049	0	0.125	1	RL higher
AUC	0.9984	0.9975	0.0009	1	0.500	0.66667	RL higher

**Table 9:** Class-wise precision, recall, and F1-score for Dataset 1 (pooled five-fold confusion matrix with and without RL).

Condition	Class	Precision	Recall	F1-Score
MLP + RL	Benign	0.9940	0.9900	0.9920
MLP + RL	Ransomware	0.9900	0.9940	0.9920
MLP (no RL)	Benign	0.9919	0.9820	0.9869
MLP (no RL)	Ransomware	0.9822	0.9920	0.9871

**Table 10:** Component-level end-to-end efficiency breakdown of the proposed residual MLP.

Component	Time (s)	RAM (MB)	Execution Role
Image loading and preprocessing	7.015	730.035	One-time image loading, resizing, and preprocessing
EfficientNetB0 feature extraction	76.968	64.668	One-time frozen CNN embedding extraction
InceptionV3 feature extraction	115.605	228.125	One-time frozen CNN embedding extraction
Feature fusion	0.009	0.000	One-time concatenation of EfficientNetB0 and InceptionV3 embeddings
Residual MLP training without RL	51.693	87.672	Mean per-fold residual MLP training using uniform sample contribution
Residual MLP inference	0.6054	1.7414	Mean per-fold held-out inference
Estimated end-to-end training pipeline without RL	251.290	730.035	One-time image-to-embedding pipeline plus mean non-RL MLP training fold
Residual MLP training with RL	107.620	138.070	Mean per-fold training; includes probe MLP training, Q-table update, final sample weighting, and final MLP training
Residual MLP inference	0.4442	0.1430	Mean per-fold held-out inference
Estimated end-to-end training pipeline with RL	307.217	730.035	One-time image-to-embedding pipeline plus mean corrected RL training fold

Note: Image-to-embedding pipeline = 7.015 + 76.968 + 115.605 + 0.009 = 199.597 s. Without RL end-to-end training pipeline = 199.597 + 51.693 = 251.290 s. With RL end-to-end training pipeline = 199.597 + 107.620 = 307.217 s.

**Table 11:** Model-wise end-to-end efficiency comparison.

Model	Configuration	Shared Image-to-Embedding Time (s)	Mean Training Time (s)	Mean Inference Time (s)	End-to-End Training Pipeline Time (s)	Shared Image-to-Embedding RAM (MB)	Mean Training RAM (MB)	Mean Inference RAM (MB)
**MLP**	**With RL**	**199.597**	**107.62**	**0.4442**	**307.217**	**730.035**	**138.07**	**0.1430**
	**Without RL**	**199.597**	**51.693**	**0.6054**	**251.290**	**730.035**	**87.672**	**1.7414**
ANN	With RL	199.597	12.028	0.181	211.625	730.035	8.689	0.137
	Without RL	199.597	5.658	0.208	205.255	730.035	12.975	0.523
CNN	With RL	199.597	222.282	0.4211	421.879	730.035	126.9312	0.4820
	Without RL	199.597	107.1744	0.4358	306.7714	730.035	61.1117	0.8133
RF	With RL	199.597	6.084	0.027	205.681	730.035	0.007	0.000
	Without RL	199.597	2.996	0.027	202.593	730.035	0.063	0.000
XGBoost	With RL	199.597	25.620	0.007	225.217	730.035	7.938	0.001
	Without RL	199.597	15.389	0.015	214.986	730.035	27.316	0.0180
SVM	With RL	199.597	6.350	0.280	205.947	730.035	0.001	0.000
	Without RL	199.597	2.557	0.354	202.154	730.035	0.045	0.000
LR	With RL	199.597	0.858	0.0095	200.455	730.035	12.198	1.015
	Without RL	199.597	0.541	0.0103	200.138	730.035	16.429	2.031

Note: The shared image-to-embedding stage (199.597 s, 730.035 MB) is identical for all models and is not repeated per classifier.

**Table 12:** Grad-CAM-based top-10 feature mapping for Dataset 1.

Sample Type	Rank	Feature	Grid Position (Row, Column)	Grad-CAM Activation
Benign	1	Feature 23	23 (3, 3)	1.0000
	2	Feature 13	13 (2, 3)	0.7458
	3	Feature 22	22 (3, 2)	0.7155
	4	Feature 24	24 (3, 4)	0.5599
	5	Feature 12	12 (2, 2)	0.5418
	6	Feature 14	14 (2, 4)	0.3887
	7	Feature 28	28 (3, 8)	0.3817
	8	Feature 18	18 (2, 8)	0.3755
	9	Feature 91	91 (10, 1)	0.3754
	10	Feature 17	17 (2, 7)	0.3523
Ransomware	1	Feature 100	100 (10, 10)	1.0000
	2	Feature 18	18 (2, 8)	0.8254
	3	Feature 28	28 (3, 8)	0.8228
	4	Feature 8	8 (1, 8)	0.8032
	5	Feature 1	1 (1, 1)	0.7574
	6	Feature 38	38 (4, 8)	0.7069
	7	Feature 9	9 (1, 9)	0.6637
	8	Feature 19	19 (2, 9)	0.6471
	9	Feature 48	48 (5, 8)	0.6461
	10	Feature 7	7 (1, 7)	0.6371

**Table 13:** Grid-order sensitivity analysis: canonical grid vs. five randomly permuted grid configurations, RL-guided residual MLP (mean ± SD across five folds).

Representation	Accuracy	Precision	Recall	F1-Score	AUC	Observation
Canonical 10 × 10 grid	0.9920 ± 0.0045	0.9901 ± 0.0069	0.9940 ± 0.0089	0.9920 ± 0.0045	0.9984 ± 0.0022	Original deterministic layout
Random shuffle 01	0.9890 ± 0.0042	0.9843 ± 0.0111	0.9940 ± 0.0055	0.9891 ± 0.0041	0.9972 ± 0.0037	Minor reduction
Random shuffle 02	0.9840 ± 0.0114	0.9750 ± 0.0231	0.9940 ± 0.0055	0.9843 ± 0.0109	0.9968 ± 0.0030	Largest reduction
Random shuffle 03	0.9880 ± 0.0045	0.9881 ± 0.0082	0.9880 ± 0.0084	0.9880 ± 0.0045	0.9972 ± 0.0036	Small reduction
Random shuffle 04	0.9880 ± 0.0097	0.9825 ± 0.0182	0.9940 ± 0.0089	0.9881 ± 0.0095	0.9939 ± 0.0105	Small reduction
Random shuffle 05	0.9890 ± 0.0074	0.9844 ± 0.0161	0.9940 ± 0.0089	0.9891 ± 0.0073	0.9970 ± 0.0044	Near-canonical performance

**Table 14:** Aggregate comparison: canonical grid vs. randomly permuted grids (five permutations, pooled), RL-guided residual MLP.

Representation	Accuracy	F1 Score	AUC	Δ Accuracy
Canonical 10 × 10 grid	0.9920 ± 0.0045	0.9920 ± 0.0045	0.9984 ± 0.0022	0.0000
Randomly shuffled 10 × 10 grid, five permutations	0.9876 ± 0.0075	0.9877 ± 0.0073	0.9964 ± 0.0054	−0.0044

**Table 15:** EldeRan predictive performance under the corrected fold-local RL protocol (mean ± SD across five folds).

Condition	RL Setting	Accuracy (Mean ± Std)	Precision (Mean ± Std)	Recall (Mean ± Std)	F1 Score (Mean ± Std)	AUC (Mean ± Std)
EldeRan_ALL	With RL	0.9036 ± 0.0125	0.9054 ± 0.0166	0.9018 ± 0.0285	0.9033 ± 0.0136	0.9633 ± 0.0049
	Without RL	0.8959 ± 0.0192	0.9009 ± 0.0178	0.8898 ± 0.0359	0.8950 ± 0.0210	0.9621 ± 0.0072
EldeRan_MI100	With RL	0.9208 ± 0.0218	0.9101 ± 0.0159	0.9346 ± 0.0326	0.9218 ± 0.0218	0.9813 ± 0.0079
	Without RL	0.9139 ± 0.0241	0.9099 ± 0.0230	0.9191 ± 0.0534	0.9138 ± 0.0264	0.9781 ± 0.0073

**Table 16:** 95% confidence intervals (t-distribution, df = 4) for the RL-guided and non-RL residual MLP on the EldeRan_ALL and EldeRan_MI100 configurations, computed from the fold-level mean and standard deviation reported in Table 15.

Condition	Configuration	Metric	Mean	Std	95% CI (t, df = 4)
EldeRan_ALL	With RL	Accuracy	0.9036	0.0125	[0.8881, 0.9191]
		Precision	0.9054	0.0166	[0.8848, 0.9260]
		Recall	0.9018	0.0285	[0.8664, 0.9372]
		F1-score	0.9033	0.0136	[0.8864, 0.9202]
		AUC	0.9633	0.0049	[0.9572, 0.9694]
EldeRan_ALL	Without RL	Accuracy	0.8959	0.0192	[0.8721, 0.9197]
		Precision	0.9009	0.0178	[0.8788, 0.9230]
		Recall	0.8898	0.0359	[0.8452, 0.9344]
		F1-score	0.8950	0.0210	[0.8689, 0.9211]
		AUC	0.9621	0.0072	[0.9532, 0.9710]
EldeRan_MI100	With RL	Accuracy	0.9208	0.0218	[0.8937, 0.9479]
		Precision	0.9101	0.0159	[0.8904, 0.9298]
		Recall	0.9346	0.0326	[0.8941, 0.9751]
		F1-score	0.9218	0.0218	[0.8947, 0.9489]
		AUC	0.9813	0.0079	[0.9715, 0.9911]
EldeRan_MI100	Without RL	Accuracy	0.9139	0.0241	[0.8840, 0.9438]
		Precision	0.9099	0.0230	[0.8813, 0.9385]
		Recall	0.9191	0.0534	[0.8528, 0.9854]
		F1-score	0.9138	0.0264	[0.8810, 0.9466]
		AUC	0.9781	0.0073	[0.9690, 0.9872]

**Table 17:** Paired Wilcoxon signed-rank test comparing fold-local RL and non-RL residual MLP on EldeRan MI top-100 (n = 5 paired folds).

Metric	With-RL Mean	Without RL Mean	Mean Difference (RL − Non-RL)	Wilcoxon W	*p*-Value	Rank-Biserial r	Direction
Accuracy	0.9208	0.9139	0.0069	1.0	0.125	1.0000	RL higher
Recall	0.9346	0.9191	0.0155	0.0	0.250	1.0000	RL higher
F1-score	0.9218	0.9138	0.0080	1.0	0.250	1.0000	RL higher
AUC	0.9813	0.9781	0.0032	1.0	0.125	0.8667	RL higher

**Table 18:** Class-wise precision, recall, and F1-score for EldeRan MI top-100 with and without RL-guided sample weighting (pooled five-fold confusion matrix).

Condition	Class	Precision	Recall	F1-Score
EldeRan_MI100 + RL	Benign	0.9327	0.9071	0.9197
EldeRan_MI100 + RL	Ransomware	0.9095	0.9346	0.9219
EldeRan_MI100 without RL	Benign	0.9183	0.9088	0.9135
EldeRan_MI100 without RL	Ransomware	0.9099	0.9191	0.9138

**Table 19:** EldeRan end-to-end computational efficiency (mean per-fold values).

Condition	RL Setting	Loading/Preparation Time (s)	Mean Train Time (s)	Mean Inference Time (s)	Preprocessing Max RAM (MB)	Mean Train RAM (MB)	Mean Inference RAM (MB)
EldeRan_ALL	With RL	33.448	111.5346	0.3454	687.848	103.4784	1.3968
	Without RL	33.448	55.8762	0.3978	687.848	83.5766	3.4970
EldeRan_MI100	With RL	33.448	109.0762	0.3514	667.246	117.4806	0.6210
	Without RL	33.448	56.0550	0.4408	667.246	61.9984	1.4224

**Table 20:** Grad-CAM top-10 feature mapping, EldeRan MI top-100 feature mapping.

Sample Type	Rank	Feature	Grid Position (Row, Column)	Grad-CAM Activation
Benign	1	EldeRan_col_13898	(10, 1)	1.000000
	2	EldeRan_col_13956	(10, 2)	0.915738
	3	EldeRan_col_14117	(10, 3)	0.736508
	4	EldeRan_col_11352	(9, 1)	0.506671
	5	EldeRan_col_11578	(9, 2)	0.425931
	6	EldeRan_col_11586	(9, 3)	0.315646
	7	EldeRan_col_106	(2, 1)	0.265248
	8	EldeRan_col_180	(3, 1)	0.249523
	9	EldeRan_col_9	(1, 1)	0.235859
	10	EldeRan_col_84	(1, 8)	0.189545
Ransomware	1	EldeRan_col_106	(2, 1)	1.000000
	2	EldeRan_col_9	(1, 1)	0.984745
	3	EldeRan_col_180	(3, 1)	0.884667
	4	EldeRan_col_29768	(10, 10)	0.724268
	5	EldeRan_col_84	(1, 8)	0.593462
	6	EldeRan_col_24	(1, 2)	0.583141
	7	EldeRan_col_107	(2, 2)	0.578005
	8	EldeRan_col_86	(1, 9)	0.545094
	9	EldeRan_col_169	(2, 8)	0.541687
	10	EldeRan_col_1769	(4, 7)	0.537124

**Table 21:** Contextual comparison of TL-RL-FusionNet with representative ransomware detection methods. The comparison is based on reported feature evidence, model design, evaluation metrics, and computational information, where available. Because the listed studies used different datasets, feature representations, execution environments, and evaluation protocols, the results are not directly comparable. N/R indicates that the data were not reported in the corresponding study.

Ref.	Feature Type	Feature Extractor	RL-Based Learning	Classifier/Model	Accuracy (%)	Training Time (s)	Prediction Time (s)	Inference Memory (MB)	FP (%)	FN (%)
[13]	Grayscale images	DenseNet-121	No	XGBoost	98.58	N/R	N/R	N/R	N/R	N/R
[14]	Binary images	VGG-16 + ResNet50	No	Bi-KNN	99.00	18.928	N/R	N/R	N/R	N/R
[12]	Binary images	VGG16, VGG19, ResNet50, InceptionV3	No	MLP	98.55	N/R	0.471	N/R	N/R	N/R
[15]	Grayscale images	VGG-16	No	SVM	92.97	N/R	N/R	N/R	N/R	N/R
[20]	Android permissions and network-traffic	N/A	Yes	A2C, DQN, DDQN, PPO	89.78	168.27	0.76	N/R	N/R	N/R
[31]	Dynamic features	N/A	Yes	FL + RL	98.00	N/R	N/R	N/R	N/R	N/R
**Our proposed method: Dataset 1**	**Behavioural feature-grid images**	**EfficientNetB0 + InceptionV3**	**Yes**	**RL-guided residual MLP**	**99.20**	**107.62**	**0.444**	**0.143**	**1.00**	**0.60**
**Our proposed method: EldeRan MI top-100**	**Public behavioural benchmark feature-grid images**	**EfficientNetB0 + InceptionV3**	**Yes**	**RL-guided residual MLP**	**92.08**	**109.0762**	**0.3514**	**0.6210**	**9.29**	**6.54**

## Data Availability

The dataset and supporting code are publicly available on GitHub at https://github.com/rande-code/TL-RL-FusionNet-Dataset/tree/master (accessed on 22 July 2026).
